# Cell Membrane-Coated Mimics: A Methodological Approach for Fabrication, Characterization for Therapeutic Applications, and Challenges for Clinical Translation

**DOI:** 10.1021/acsnano.1c03800

**Published:** 2021-10-26

**Authors:** Vaishali Chugh, K. Vijaya Krishna, Abhay Pandit

**Affiliations:** CÚRAM, SFI Research Centre for Medical Devices, National University of Ireland Galway, Galway H91 W2TY, Ireland

**Keywords:** cell membrane, template, biomimetic, biointerfacing, drug delivery, personalized medicine, tumor microenvironment, good manufacturing practice, detoxification, therapeutic applications

## Abstract

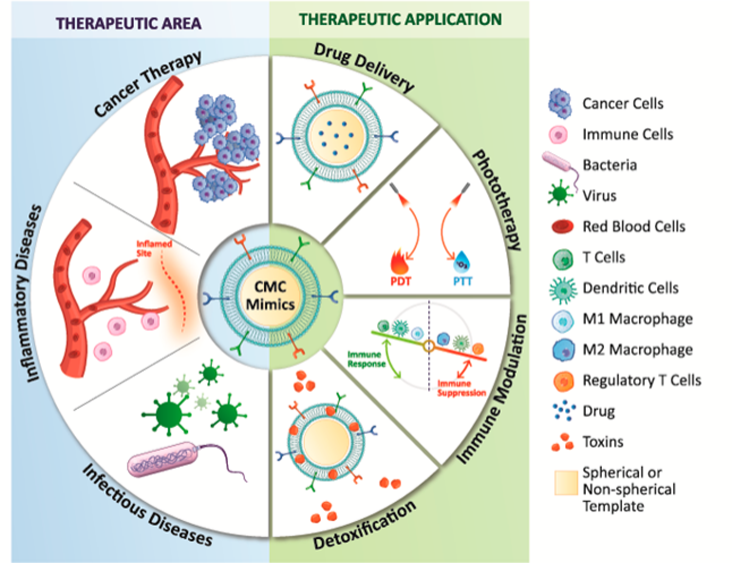

Cell membrane-coated (CMC) mimics are micro/nanosystems that combine an isolated cell membrane and a template of choice to mimic the functions of a cell. The design exploits its physicochemical and biological properties for therapeutic applications. The mimics demonstrate excellent biological compatibility, enhanced biointerfacing capabilities, physical, chemical, and biological tunability, ability to retain cellular properties, immune escape, prolonged circulation time, and protect the encapsulated drug from degradation and active targeting. These properties and the ease of adapting them for personalized clinical medicine have generated a significant research interest over the past decade. This review presents a detailed overview of the recent advances in the development of cell membrane-coated (CMC) mimics. The primary focus is to collate and discuss components, fabrication methodologies, and the significance of physiochemical and biological characterization techniques for validating a CMC mimic. We present a critical analysis of the two main components of CMC mimics: the template and the cell membrane and mapped their use in therapeutic scenarios. In addition, we have emphasized on the challenges associated with CMC mimics in their clinical translation. Overall, this review is an up to date toolbox that researchers can benefit from while designing and characterizing CMC mimics.

Pharmacological/drug-based therapies are the most common and foremost recourse prescribed for treating diseases and disorders in the human body. In practice, for many years, these therapies have improved health and extended lives without the need for aggressive interventions.^1−6^ However, the advent of nanomedicine has revolutionized this traditional approach for disease diagnosis and treatment. Nanomedicine combines the principles of nanotechnology, immunology, and biomaterials to create delivery systems with significantly improved safety and efficacy.^7−9^

Delivery systems have two main functions: to execute a specific application that they are designed for and to interact favorably with the complex physiological environment surrounding them to support and enhance their function. Loading a drug of interest or modulating its physiochemical properties can improve these functions partially. However, it is vital to ensure that they have biointerfacing capabilities to avoid roadblocks during clinical translation.^10−12^ Biointerfacing capabilities include improving stimuli responsiveness, reducing nonspecific interactions, increasing circulation times, and evading uptake or clearance by the reticuloendothelial system.^13−15^ While PEGylation offered some respite by introducing stealth properties, minimizing nonspecific interactions and prolonging circulation, yet negative immunogenic response and allergic reactions were unavoidable.^16,17^ An alternative approach is incorporating ligands (antibodies,^18,19^ aptamers,^20,21^ peptides,^22,23^ and small molecules^24,25^) to improve target efficacy, but this rendered the system overly complicated for scale-up. These strategies were only partial remedies and not universally applicable or sufficient for clinical translation.

Vital clues to improve the biointerfacing capabilities of synthetic delivery systems can be obtained by understanding the structure, function, and homeostasis of cells in the complex physiological environment surrounding them. Incorporating cell properties like shape, flexibility,^26,27^ compartmentalization,^28−30^ lipid bilayer structure,^31,32^ autonomous and specific functionality,^33−35^ and protecting cargo^36,37^ can be advantageous in delivery systems. In this regard, researchers have attempted to use liposomes,^38,39^ polymeric micelles,^40^ or naturally occurring extracellular vesicles^41^ as delivery systems. For example, Doxil and GenexoltPM are the first FDA-approved liposomal and polymeric micelle formulations, respectively, translated into the clinic and many more under different phase trials.^42,43^ However, liposome and polymeric micelle’s long-term stability issues, degradation during sterilization, and complex surface modifications for active targeting still remain a challenge for large scale-up.^42−46^ For avoiding surface modification complexity, extracellular vesicles are viable alternatives as a delivery system. These are lipid bilayer vesicles, naturally secreted by the cells that display the same proteins, ligands, and targeting moieties like a cell on its surface.^47^ Unfortunately, the existing isolation and purification methods for vesicle production cause functional heterogenicity and low yield.^48−50^ Besides, low drug loading efficiency also limits their use for a wide range of applications.^51−53^ The cell membrane is a major structural component of a cell and extracellular vesicles and replicates their surface functionality. If done correctly, the cell membrane conserves this functionality post-isolation, and its coating improves biointerfacing capabilities. Referred to as cell membrane-coated (CMC) mimics henceforth, these intelligently engineered delivery systems combine the biomimetic features of the cell membrane and the functional versatility of a template. The template (spherical or nonspherical) acts as the central scaffold that carries a payload of interest and provides a structural basis. The cell membrane offers surface functionality that mimics a natural cell to improve accumulation and efficacy at the target site.^54^ Their assembly process utilizes noncovalent interactions and physical and soft techniques and eliminates a need for complex chemical processing and traditional synthetic modifications.^55−57^ Compared to the conventional delivery systems, these CMC mimics demonstrate excellent biological compatibility, stealth properties, and retain cellular properties for active targeting using receptor–ligand interactions.^58−66^

In this review, we focus on providing a detailed insight into the various aspects of designing CMC mimics. We begin with an overview of different cell types, their inherent biological properties, and suitability for specific therapeutic applications including cancer, inflammatory diseases, infectious diseases, and their potential use in personalized medicine. In the next section, we present protocols for isolating cell membranes from both nucleus-free and nucleus-containing cells with minimal nuclear and mitochondrial contamination. It is vital to follow protocols that conserve their surface functionality and mechanical stability during the isolation process. Next, we present an overview of templates and their properties available for cell membrane coating. Selecting the right template allows for the chemical and genetic tunability of the mimics and improves bioimaging,^67,68^ drug delivery,^55,69,70^ diagnostic,^71−74^ biosensing,^75^ detoxification,^76,77^ and phototherapy performance.^56,78,79^ We then highlight the processes used for CMC assembly and the challenges for large-scale production, followed by physiochemical and biological characterization techniques that validate their structural integrity and functionality. The last part of the review presents examples of CMC mimics designed for therapeutic applications and *in vitro* and *in vivo* models that evaluate their efficacy. Finally, we conclude with an overview of current challenges en route to clinical translation.

## Biological Properties of Different Cell Membranes in CMC Mimics

The cell membrane is the outermost protective layer of a cell with a thickness of around 5–10 nm, mainly composed of lipids, proteins, and carbohydrates, and it interacts and performs complex biological functions with the surrounding environment for survival and proliferation.^80,81^ Bilayer assembly of lipids incorporates structural rigidity and fluidity,^82^ while carbohydrates are responsible for cellular recognition,^83,84^ and proteins play a vital part in signaling and adhesion, briefly.^85^ The composition and properties of these three components of the cell membrane differentiate them. The possibility of benefiting from native functionalities originating from cell membranes has resulted in significant research interest in CMC mimics.^86−89^

**Figure 1:**
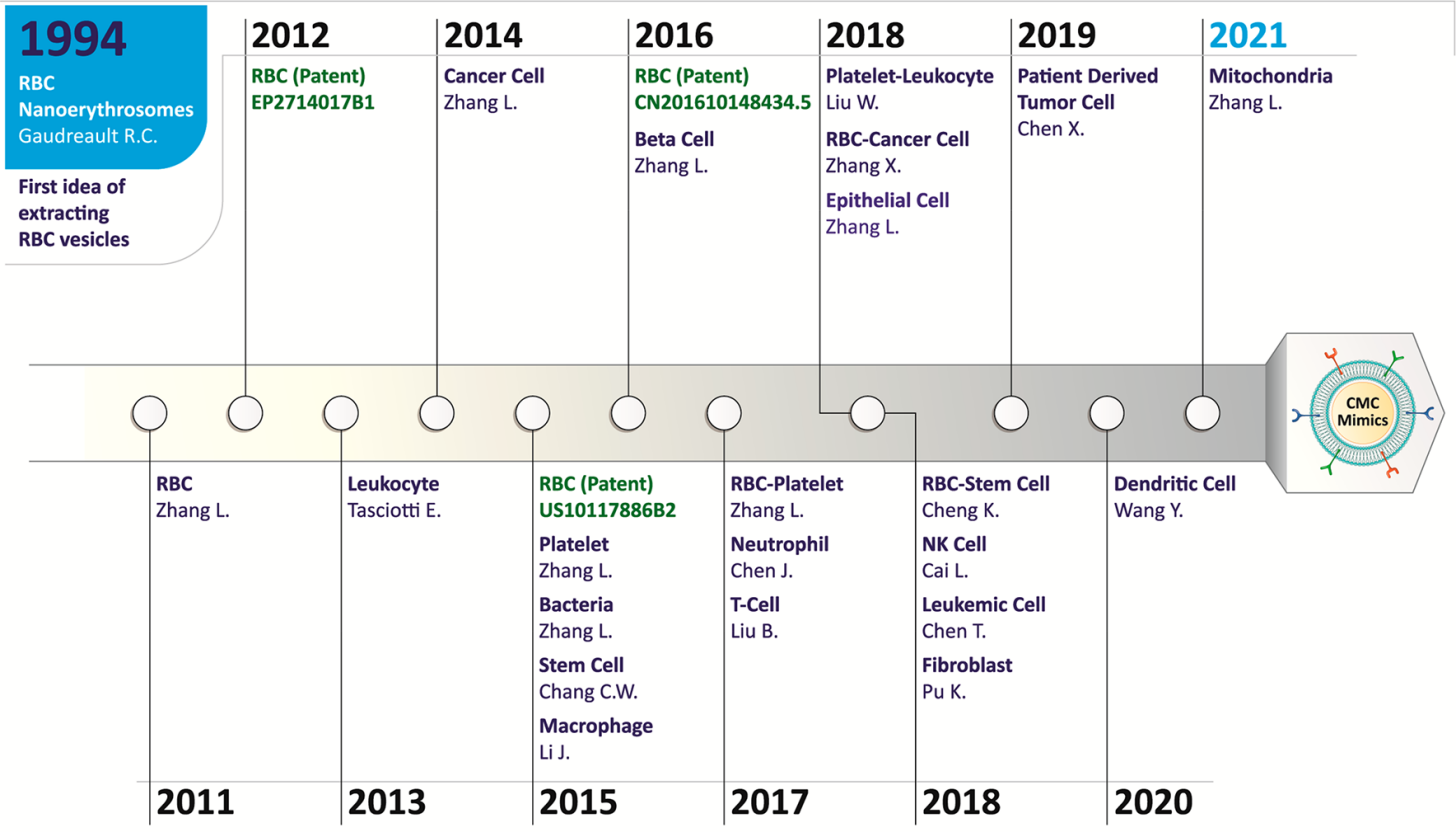
Timeline of different cell sources utilized in CMC mimics fabrication. The idea of isolating the RBC vesicles was reported in 1994^90^ and gained significant research interest in utilizing cell membrane vesicles for coating onto a template to design CMC mimics in 2011.^62^ For designing these mimicking systems, the cell membrane from a wide variety of cells source (leukocyte,^63^ cancer cell,^92^ platelet,^93^ bacteria,^94^ stem cell,^95^ macrophage,^69^ β-cell,^96^ RBC-platelet hybrid,^97^ neutrophil,^55^ T-cell,^98^ platelet-leukocyte hybrid,^99^ RBC-cancer cell hybrid,^100^ epithelial cell,^101^ RBC-stem cell hybrid,^86^ natural killer (NK) cell,^102^ leukemic cell,^103^ fibroblast,^104^ patient-derived tumor cell,^105^ dendritic cell^106^) has been explored depending upon the importance of cells for a specific application. Recently, intracellular organelle membrane coating was investigated using mitochondria as a model organelle. These CMC mimics have shown great potential for use in personalized medicine.^92^ Some patents granted on these CMC mimics using the RBC membrane are highlighted in green in this figure.

Figure 1 provides a timeline of different cell sources utilized in the CMC mimics fabrication. The idea of isolating RBC vesicles was explored in 1994^90^ and gained significant research interest in utilizing cell membrane vesicles for coating onto a template to design CMC mimics in 2011.^62^ Until 2020, the natural cell membrane has widely been used from different cell types, but recently, the outer intracellular membrane from the mitochondria has also been explored to enhance biointerfacing capabilities.^91^ This section describes the specific biological function of the cell membrane of various cell types and the intracellular organelle that they offer to a CMC mimic.

### Red Blood Cell Membrane

Red blood cells (RBCs) are the most abundant cell type of the human body, with the longest circulation time of approximately 120 days.^107^ RBCs transmembrane express protein cluster of differentiation 47 (CD47), also known as the ‘do not eat me’ marker,^108^ selectively binds to signal-regulatory protein alpha (SIRPα) glycoprotein expressed by macrophages to prevent its uptake.^109,110^ RBCs are also responsible for oxygen transport to various tissues and organs in the body^111^ and are involved in pathogen removal by oxycytosis.^112^ Their membrane is rich in glycophorins that attract pathogens to their surface to release oxygen for killing them.^113^ Thus, coating the template with an RBC membrane improves long-term circulation,^62^ pathogens removal,^64,114^ and toxins absorption^115,77^ for detoxification applications. These specific advantages have popularized the use of RBC membrane-coated CMC mimics.

### Platelet Cell Membrane

Platelets, also known as thrombocytes, inhibit bleeding by forming clots and help in tissue repair.^116^ Platelets membrane like RBCs express CD47 receptor proteins on their surface that help in evading macrophages. Additional membrane proteins on platelets: integrin like αIIbβ3, α6β1, and P-selectin help in targeting tumor cells,^117^ glycoprotein Ib (GPIb/IX/V) complex in binding to exposed subendothelial collagen at the injury site in blood vessels by interacting with von Willebrand factor (VWF),^117^ clusters of differentiation 55 (CD55), and clusters of differentiation 59 (CD59) for immune modulation,^118^ toll-like receptors for pathogen removal.^119^ Platelets are involved in a cross-talk with inflamed endothelium cells and bind with immune cells to redirect them to the injury site.^120^ Thus, coating the template with a platelet membrane offers an escape from macrophage detection, selective adhesion to tumor tissues or injured vessels,^121,70^ targeting of vascular disorders,^93,122,123^ and binding ability to circulatory tumor cells^87^ and pathogen removal.^93^

### Macrophage Cell Membrane

Macrophages are part of the innate immune system, known for removing unwanted or foreign materials/bacteria/viruses from the human body by engulfing them (phagocytosis) using recognition receptors such as scavenger receptors, mannose receptors, and toll-like receptors ((TLR)-2, -4, -5).^124,125^ Derived from circulatory monocytes, they are present in all the tissues. During infections or tissue damage, cytokines actively recruit monocytes where they differentiate into macrophages.^126^ Chemokine receptors on the macrophage membrane like C–C chemokine receptors type 2 (CCR2), C–X–C chemokine receptor type 1 (CXCR1), C–C chemokine receptor type 7 (CCR7), *etc.*, facilitate their recruitment at the inflammation site.^127^ Along with other leukocytes, macrophage membranes also express adhesions molecules like P-selectin glycoprotein ligand-1 (PSGL-1), L-selectin, lymphocyte function-associated antigen 1 (LFA-1), L-selectin, and very late antigen-4 (VLA-4) that assist in their recruitment and cell adhesion.^128,129^ Thus, coating the template with the macrophage membrane has the potential to bind pathogens and can also easily escape from macrophage detection to provide active targeting at inflammatory sites^130^ and tumors.^69,131,132^

### Neutrophil Cell Membrane

Neutrophils belong to the innate immune system and constitute around 40–60% of the white cell population in a healthy human body.^133^ In response to inflammation, their production rate in bone marrow increases by at least 10-fold.^134^ After leaving the bone marrow, their targeting abilities depend on their phenotypic changes and surface. Neutrophils are usually resting when circulating in healthy body receptors.^135,136^ They become activated by cytokines or chemokines like tumor necrosis factor-alpha (TNF-α), granulocyte-macrophage colony-stimulating factor (GM-CSF), interleukin 8 (IL-8), and interferon gamma (IFN-γ) which mobilize them to the infection or inflammation site.^137^ Conformational changes in integrin adhesion receptors like very late antigen-4 (VLA-4), lymphocyte function-associated antigen 1 (LFA-1), macrophage-1 antigen (Mac-1), P-selectin glycoprotein ligand-1 (PSGL-1), and L-selectin also facilitate neutrophil migration through extravasation from blood vessels.^133,138^ Thus, coating the template with the activated neutrophil membrane actively targets the tumors^139,55^ and inflammatory sites.^66^

### Natural Killer Cell Membrane

Natural killer (NK) cells are part of the innate immune system and the first line of defense against tumor and virally infected cells that do not require any prior activation like other immune cells (T cells, B cells).^140^ In human peripheral blood, the NK cells comprise 10–15% of the total lymphocyte population. These cells contain many activating and inhibitory receptors on their surface that selectively target tumor/virally infected cells without affecting healthy cells.^141^ Some of the important activating receptors are NK group 2D (NKG2D), DNAX accessory molecule-1 (DNAM-1), natural cytotoxicity receptor (NKp30), *etc.*;^142^ integrin adhesion receptors are LFA-1, VLA-4, Mac-1, PSGL-1, and L-selectin (along with other leukocytes), *etc.*, that help in extravasation from blood vessels.^142,143^ These cells also activate other immune cells like T cells by releasing cytokines and chemokines.^144^ NK cell lines, for example, KHYG-1 and NK-92 membranes, also contain activating and adhesion receptors like primary NK cell membrane, facilitating their use in clinical trials.^145−147^ These cell lines are also easy to culture and expand *in vitro.* Therefore, utilizing NK cell line membrane in CMC mimics could also be a potential alternative. Recently, chimeric antigen receptor (CAR)-NK and CAR-NK-92 technologies began undergoing clinical trials for immunotherapy.^148,149^ Thus, coating the template with the NK cell membrane has the potential to actively target inflammation, infection, and tumor sites without prior activation.^102,150^

### T-Cell Membrane

T cells are part of the adaptive immune system that can recognize antigens using T-cell receptors (TCR).^151^ TCRs cannot bind to antigens directly and require peptides fragments of antigens for binding. These fragments are presented to them by major histocompatibility complex molecules (MHC I or II) present on antigen-presenting cells (dendritic cells or macrophages).^152^ Naive T cells recognize these specific fragments and differentiate into subsets like cytotoxic, helper, or regulatory T cells. Cytotoxic T cells express cluster of differentiation 8 (CD8) coreceptor (CD8^+^ T cell) that recognizes antigens on MHC-I molecules and can kill the infected cells (virus/bacteria/cancer cells) by releasing cytotoxic granules or Fas/FasL interaction.^153^ Helper T cells express cluster of differentiation 4 (CD4) coreceptor (CD4^+^ T cells) recognize antigens on MHC-II molecules and regulate immune response that indirectly affects the infected cells.^154^ According to literature reports, helper T cells play an important role in treating HIV due to its high-affinity receptor (CD4^+^ T).^155^ CAR-T cell therapy is an FDA-approved therapy for multiple myeloma (ABECMA) and is under evaluation for treating other cancer types and avoid unwanted side effects.^156^ Therefore, utilizing the T-cell membrane in CMC mimics could be a potential strategy for treating cancer and infectious diseases.^98,157−159^

### Dendritic Cell Membrane

Dendritic cells (DCs) are central players of the immune system that link innate and adaptive immune systems. These cells are also known as “professional” antigen-presenting cells (APCs).^160^ DCs are the first immune cells to become activated in the human body post a pathogenic attack (bacteria, virus, or cancer cells).^161,162^ Even in their resting immature state, iDCs are involved in phagocytosis. They encapsulate pathogens and process them, degrade them into fragments, and present them on the MHC molecules on their surface.^163^ During this activation process, iDCs mature and migrate to adaptive immune cells (T cells and B cells) and present antigens for their activation. During antigen presentation, DCs upregulate the expression of co-stimulatory receptors molecules CD86, CD83, CD80, and CD40 on their cell membrane.^164^ These molecules effectively bind to their corresponding receptors on T cells and trigger the release of cytokines (interleukin, IL-12 or IL-10) from DCs that differentiate T cells into their pro-inflammatory or anti-inflammatory subsets. According to experimental reports, one mature DC can stimulate up to 100–3000 T cells.^165,166^ Thus, CMC mimics fabricated with mature dendritic cell membrane can generate sufficient immune response to activating T cells, required to treat several tumors and infectious diseases.^106,167,168^

### Cancer Cell Membrane

Cancer cells can escape the immune system and are known for their rapid and infinite proliferation. Because of their robust nature, it is easy to culture and expands them *in vitro*. Different types of cancer cell membranes express numerous tumor-specific antigens and adhesion molecules on their surface. Some of them include cadherins, integrins, galectin-3, lymphocyte-homing receptors (like clusters of differentiation 44 (CD44)), epithelial adhesion molecules, and mucoprotein-1 that play a vital role in cell-to-cell and cell-to-matrix interactions.^169−171^ Mainly, cancer cell membranes have self-targeting abilities to adhere to their homologous cells.^65,172^ Thus, coating a template with the cancer cell membrane allows it to escape from macrophage detection and for homotypic tumor targeting^173−175^ and helps in the development of personalized medicine for cancer.^105^

### Stem Cell Membrane

Stem cells are known for their ability to replicate indefinitely and differentiate into specialized cell types in the body. Among other stem cells, mesenchymal stem cell (MSC)-based therapies have shown immense potential as regenerative medicine^176^ and have entered many clinical trials.^177,178^ These cells can specifically target different cancerous and metastatic diseases because of their intrinsic tumor tropic properties,^179−181^ they are readily isolated, are stable through multiple *in vitro* passages, and are produced under good manufacturing practice (GMP) conditions.^182,183^ Various chemokines and cytokine receptors like CCR1, CCR2, CXCR1, CXCR2, *etc.*, help the MSCs to migrate to the inflammatory or injured site.^184^ Like leukocytes, stem cells also undergo rolling, adhesion, and an extravasation process. Thus, coating the template with a stem cell membrane provides actively targeting abilities toward tumor^95,185,186^ and degenerative diseases.^187,188^

### Bacterial Cell Membrane

Bacteria have an additional peptidoglycan cell wall, unlike other mammalian cell types. Gram-positive bacteria have a thick peptidoglycan cell wall and no outer membrane, while Gram-negative bacteria have thin cell walls as well as lipopolysaccharide outer membranes.^189^ Both the Gram-positive and Gram-negative bacteria secrete membrane vesicles. Gram-positive bacteria secrete extracellular vesicles (EVs), whereas Gram-negative bacteria secrete outer membrane vesicles (OMVs).^190^ These membrane vesicles express several immunogenic antigens with adjuvant properties and pathogens-associated patterns that help immune modulation.^94,191^ Thus, coating the template with the bacterial membrane vesicles (*Escherichia coli* (*E. coli*); *Staphylococcus aureus* (*S. aureus*); *Klebsiella pneumonia* (*K. pneumonia*)) provides an antibacterial immune response,^94^ vaccination against bacterial infection,^94,192,193^ and tumor targeting abilities.^194,195^

### Hybrid Cell Membrane

The hybrid cell membrane coating strategy fuses cell membranes from multiple cell types to incorporate multiple cell-specific functional properties in a single mimic.^168,196,197^ For example, CMC mimics designed using RBC and B16-F10 melanoma cancer cell membrane express both CD47 transmembrane protein from RBCs and self-recognition markers (glycoprotein, gp100) from the cancer cell membrane.^100,198^ Overall, these RBC-cancer hybrid membranes provide several features like long-term circulation, immune evasion, and homotypic targeting abilities in the CMC mimics.^196^ Depending on the specific target application, the relative amount of each membrane can be varied for designing CMC mimics. Thus, hybrid membrane coating by coupling different cell types (refer to previous sections) provides the possibility of designing CMC mimics with multiple desired functionalities, thus offering several advantages in various therapeutic applications.^97,99,199−201^

### Intracellular Cell Membrane (Organelle)

Intracellular membranes from organelles of eukaryotes display the same fundamental structure as the plasma membrane, with the phospholipid bilayer responsible for specific functions.^202^ Targeting intracellular membrane functions can be an intelligent strategy for treating several diseases. For example, the delivery of biomolecules across nuclear membranes is considered safe and effective gene therapy.^203,204^ For drug-resistant bacterial or viral infections, it is preferable to block the alteration of intracellular membranes with pathogens and inhibit their intracellular replication.^205^ Inducing permeability in the mitochondrial, nucleus, and lysosomal membranes is a well-established strategy to overcome drug resistance during cancer treatment.^206^ Recently, CMC mimics fabricated using intracellular membranes were explored to targeted detoxification and molecular detection in ABT-263-induced thrombocytopenia.^91^ Therefore, coating templates with the intracellular membranes can be an innovative approach to probe the complex intracellular machinery for several therapeutic applications.

## Protocols for Cell Membrane Extraction

**Figure 2:**
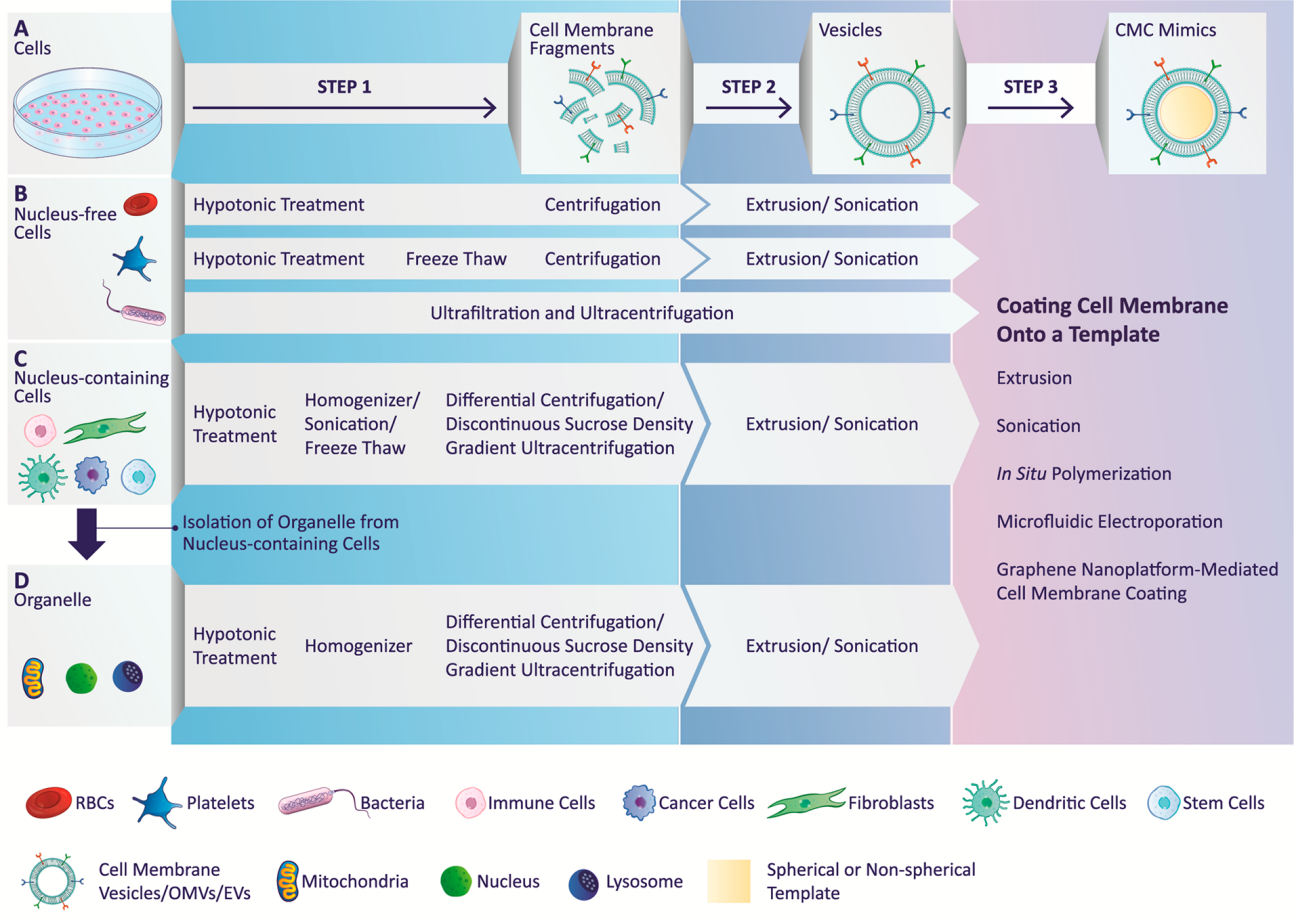
A schematic illustration of isolating and preparing membrane vesicles from nucleus-free, nucleus-containing cells, and organelle before coating: (A) The two-step process involves extracting cell membrane fragments (step 1) and preparing cell membrane vesicles (step 2). Depending on the type of cell used, cell membrane extraction and vesicle formation require a combination of techniques: (B) nucleus-free cells and (C) nucleus-containing cells. (D) Organelles: Step 3 is the final step of coating cell membrane onto a template (spherical or nonspherical) using suitable technique mentioned. Abbreviations: RBCs, red blood cells; OMVs, outer membrane vesicles; EVs, extracellular vesicles.

There are two categories of cells: nucleus-free or nucleus-containing cells. There are several reports on cell membrane isolation from various cell types. An attempt has been made to simplify the procedure and discuss the main steps involved during the isolation (Figure 2).

Cell membranes isolation protocols aim to separate the cell membrane from the cell with minimal or no nuclear/mitochondrial/cytosol contamination depending on the cell type. Using a pure cell membrane helps in assembling CMC mimics by enhancing an efficient and homogeneous surface coating with maximal functional replication on the template surface. The extraction buffers (pH 7–7.4) are supplemented with protease/phosphatase inhibitor cocktails in ice-cold conditions to protect the membrane proteins from degradation.^55,106,188,207,208^ Prior to isolation, cells are washed multiple times with 1× phosphate-buffered saline (PBS) buffer to remove remnants from cell culture media. Post-isolation, the cell membrane is lyophilized and usually stored at −80 °C to maintain the long term stability and function of membrane proteins.^175,187,209,100,173^

The cell membrane isolation mainly involves two steps depending on the cell type (Figure 2):

(1) Gentle rupturing of cells using detergent-free hypotonic treatment (osmotic imbalance) or a combination of hypotonic treatment and physical disruption technique
(2) Separation and purification of the cell membrane from intracellular components using multiple centrifugation steps, differential centrifugation, or discontinuous sucrose density gradient centrifugation.

In this section, we have discussed the membrane isolation methodology from nucleus-free, nucleus-containing cells and the recently explored intracellular organelle (mitochondria) in designing CMC mimics. All the different conditions (hypotonic buffers, physical disruption techniques, and centrifugation speeds) used in cell membrane isolation are summarized in Tables 1 and 2.

**Table 1:** Cell Membrane Isolation from Nucleus-Free Cells^a^

cell type	hypotonic buffer (ice cold)	disruption technique (ice cold)	method of isolation (4 °C)	ref
RBCs	0.25× PBS	hypotonic treatment; centrifuge 3–4 times to get pink pellet	800*g*, 5 min	(62)
	0.25× PBS, 0.2 mM EDTAK2		10,000 rpm, 5 min	(211)
	deionized water, EDTA		4000 rpm, 10 min	(210)
			4800 rpm, 20 min	
platelets	1× PBS, 1 mM EDTA, protease inhibitor cocktail	repeated freeze thaw cycles	4000*g*, 3 min	(93)
	–	7 freeze–thaw cycles and sonication	discontinuous sucrose gradient ultracentrifugation	(213)
			5%, 40%, 55%	
			cell membrane: interface between 5% and 40%	
	deionized water	3 freeze–thaw cycles	21,000*g*, 7 min	(212)

bacteria	ultrafiltration	method of OMVs or EVs isolation	ref
*E. coli*	4000*g* for 10 min, filtration through a 0.45 μm vacuum filter, concentration of filtrate using ultrafiltration with a 100 kDa amicon centrifugal filter	150,000*g*, 2 h	(94)
*S. aureus*	10,000*g* for 20 min, filtration through a 0.45 μm vacuum filter, concentration of filtrate using ultrafiltration with a 100 kDa amicon centrifugal filter	150,000*g,* 3 h	(192)
*K. pneumoniae*	3220*g* for 15 min, filtration through a 0.22 μm vacuum filter, concentration of filtrate using a 100 kDa ultrafiltration tube	150,000*g,* 1 h	(193)

a Abbreviations: RBC, red blood cells; PBS, phosphate-buffered saline; EDTA, ethylenediaminetetraacetic acid; EDTAK2, dipotassium EDTA; *E. coli*, *Escherichia coli*; *S. aureus*, *Staphylococcus aureus*; *K. pneumonia*, *Klebsiella pneumonia*; *g*, relative centrifugal force; rpm, revolutions per minute; min, minutes, h, hour; OMVs, outer membrane vesicles; EVs, extracellular vesicles.

**Table 2:** Cell Membrane Isolation from Nucleus-Containing Cells and Intracellular Organelle^a^

cell type	hypotonic buffer (ice cold)	disruption technique (ice cold)	method of isolation (4 °C)	ref
Intracellular Organelle: Mitochondria				
isolation of mitochondria from mouse liver	210 mM mannitol, 70 mM sucrose, 5 mM Tris-HCl,1 mM EDTA	Kinematic Polytron PT-2000 homogenizer (power setting 7 for 15 strokes)	2000*g*, 10 min	(91)
			7000*g*, 10 min	
			followed by sucrose density gradient ultrafiltration	
			top: 15 mL of 1.0 M sucrose solution	
			bottom: 15 mL of 1.5 M sucrose solution	
			60,000*g*, 20 min	
			mitochondria- interface between the two sucrose layers	
isolation of membrane from mitochondria	ultapure water (20 min) followed by addition of 1.4 M sucrose solution (5 min)	Kinematic Polytron PT-2000 homogenizer (power setting 7 and 30 strokes)	differential centrifugation	
			12,000*g*, 10 min	
			100,000*g*, 30 min	
Immune Cells				
neutrophils	0.5% (w/v) BSA, 75 mM sucrose, 225 mM mannitol, 0.5 mM EDTA, 30 mM Tris-HCl, protease inhibitor cocktail	Dounce homogenizer, tight-fitting pestle (50–100 passes)	differential centrifugation	(55)
			800*g*, 10 min	
			10,000*g*, 20 min	
			100,000*g*, 60 min	
	75 mM sucrose, 225 mM d- mannitol, 0.5 mM EGTA, 30 mM Tris-HCl, protease inhibitor cocktail	Dounce homogenizer, tight-fitting pestle (20 passes)	differential centrifugation	(66)
			20,000*g,* 25 min	
			100,000*g*, 35 min	
NK-92	10 mM Tris-HCl, 10 mM MgCl_2_, 1 mM KCl, 25 mM sucrose, 2 mM PMSF, 200 μg/mL trypsin chymotrypsin inhibitor, 10 μg/mL DNase, 10 μg/mL RNase	homogenized, 5 min (20 s pulse, 30 s in between pulses)	discontinuous sucrose gradient ultracentrifugation	(67, 102)
			30%, 40%, and 55% sucrose in 0.85% saline	
			cell membrane: interface between 30% and 40%	
	cell lysis buffer, protease inhibitor cocktail	water bath sonication, 20–30 min	differential centrifugation	(150)
			3500*g,* 10 min	
			20,000*g*, 25 min	
			100,000*g,* 50 min	
mouse natural killer cells	cell lysis buffer, protease inhibitor cocktail	water bath sonication, 20–30 min	differential centrifugation	(150)
			3500 *g,* 10 min	
			20,000*g*, 25 min	
			100,000*g,* 50 min	
human cytotoxic T-lymphocyte cells	10 mM Tris-HCl, 1 mM KCl, 1 mM MgCl_2_, phosphatase inhibitor cocktail	Dounce homogenizer, tight-fitting pestle (20 passes)	discontinuous sucrose gradient ultracentrifugation	(98)
			30%, 40%, and 55%	
			cell membrane: interface between 30% and 40%	
RAW264.7	10 mM Tris-HCl, 1 mM MgCl_2_	Mini-extruder without a polycarbonate membrane (20 times)	homogenate mixed with 1 M sucrose to a final concentration of 0.25 M sucrose	(129)
			centrifugations	
			2000*g*, 10 min	
			3000*g*, 30 min	
	20 mM Tris-HCl, 2 mM MgCl_2_, 10 mM KCl, protease inhibitor cocktail	Dounce homogenizer, tight-fitting pestle (20 passes)	differential centrifugation	
			3200*g*, 5 min	
			20,000*g*, 30 min	
			80,000*g*, 1.5 h	
	1 mmol/L NaHCO_3_, 0.2 mmol/L EDTA, 1 mmol/L PMSF	repeatedly grinding (20 times)	differential centrifugation	(69)
			3200*g*, 5 min	
			100,000*g*, 30 min	
THP-1	10 mM Tris-HCl, 10 mM MgCl_2_, 1 mM KCl, 25 mM sucrose, 2 mM PMSF, 200 μg/mL trypsin chymotrypsin inhibitor, 10 μg/mL DNase, 10 μg/mL RNase	Dounce homogenizer, tight-fitting pestle (20–30 passes)	discontinuous sucrose gradient ultracentrifugation	(63)
			30%, 40%, and 55% sucrose in 0.85% saline	
			cell membrane: interface between 30% and 40%	
J774	10 mM Tris-HCl, 10 mM MgCl_2_, 1 mM KCl, 25 mM sucrose, 2 mM PMSF, 200 μg/mL trypsin chymotrypsin inhibitor, 10 μg/mL DNase, 10 μg/mL RNase	Dounce homogenizer, tight-fitting pestle (20–30 passes)	discontinuous sucrose density gradient ultracentrifugation	(63)
			30%, 40%, and 55% sucrose in 0.85% saline	
			cell membrane: interface between 30% and 40%	
	75 mM sucrose, 20 mM Tris-HCl, 2 mM MgCl_2_, 10 mM KCl, protease inhibitor cocktail	Dounce homogenizer, tight-fitting pestle (20 passes)	differential centrifugation	(130)
			3200*g*, 5 min	
			20,000*g,* 25 min	
			100,000*g*, 35 min	
dendritic cells	25 mM sucrose, 10 mM Tris-HCl, 1 mM MgCl_2_, 1 mM KCl, 2 mM PMSF, protease and phosphatase inhibitor cocktail	Dounce homogenizer, tight-fitting pestle (25 passes)	differential centrifugation	(106)
			800*g,* 5 min	
			21,000*g*, 10 min	
Cancer Cells				
HeLa	membrane and cytosol protein extraction kit and PMSF	3 freeze–thaw cycles	differential centrifugation	(219)
			700*g,* 10 min	
			14,000*g*, 30 min	
MCF-7	HEPES B buffer: 2.38 g/L Hepes, 0.476 g/L MgCl_2_, 0.292 g/L EDTA, 0.154 g/L DTT, 0.746 g/L KCl, proteinase inhibitor cocktail	IKA T10 basic homogenizer (three times, four-speed position)	discontinuous sucrose density gradient ultracentrifugation	(198)
			30%, 40%, and 55%	
			cell membrane: interface between 30% and 40%	
	0.5% (w/v) BSA, 75 mM sucrose, 225 mM mannitol, 0.5 mM EDTA, 30 mM Tris-HCl, protease inhibitor cocktail	sonication	differential centrifugation	(175)
			800*g*, 10 min	
			10,000*g*, 20 min	
			100,000*g*, 60 min	
4T1	10 mM Tris, 10 mM MgCl_2_, protease inhibitor cocktail	homogenizer (22,000 rpm for 1 min)	differential centrifugation	(220, 221, and 72)
			500*g,* 10 min	
			10,000*g*, 20 min	
			100,000*g*, 60 min	
B16-F10	30 mM Tris-HCl, 75.9 mM sucrose, 225 mM d-mannitol, phosphatase inhibitor, protease inhibitor cocktail	Kinematica Polytron PT 10/35 probe homogenizer (70% power, 15 passes)	differential centrifugation	(222)
			10,000*g*, 25 min	
			150,000*g*, 35 min	
	20 mM Tris HCl, 10 mM KCl, 2 mM MgCl_2_, protease inhibitor cocktail	pipetting thoroughly	differential centrifugation	(214)
			3200*g*, 5 min	
			21,000*g*, 25 min	
			45,000*g*, 5 min	
	20 mM Tris HCl, 10 mM KCl, 2 mM MgCl_2_, protease inhibitor cocktail	Dounce homogenizer, tight-fitting pestle (20 passes)	differential centrifugation	(92)
			3200*g*, 5 min	
			20,000*g,* 25 min	
			100,000*g*, 35 min	
LL/2, CMT64.OVA, MB49, A549, SKOV-3	20 mM Tris HCl, 10 mM KCl, 2 mM MgCl_2_, protease inhibitor cocktail	pipetting thoroughly	differential centrifugation	(214)
			3200*g*, 5 min	
			21,000*g*, 25 min	
			45,000*g*, 5 min	
K562	20 mM Tris HCl, 10 mM KCl, 2 mM MgCl_2_, protease inhibitor cocktail	Dounce homogenizer, tight-fitting pestle (20 passes)	differential centrifugation	(92, 103)
			3200*g*, 5 min	
			20,000*g,* 25 min	
			100,000*g*, 35 min	
MDA-MB 435	20 mM Tris HCl, 10 mM KCl, 2 mM MgCl_2_, protease inhibitor cocktail	Dounce homogenizer, tight-fitting pestle (20 passes)	differential centrifugation	(92, 223)
			3200*g*, 5 min	
			20,000*g,* 25 min	
			100,000*g*, 35 min–1.5 h	
patient-derived tumor cell	20 mM Tris HCl, 10 mM KCl, 2 mM MgCl_2_, protease inhibitor cocktail	Dounce homogenizer, tight-fitting pestle (20 passes)	differential centrifugation	(105)
			3200*g*, 5 min	
			20,000*g,* 25 min	
			80,000*g*, 1.5 h	
DU145, CAL27, HCT116	20 mM Tris HCl, 10 mM KCl, 2 mM MgCl_2_, protease inhibitor cocktail	Dounce homogenizer, tight-fitting pestle (20 passes)	differential centrifugation	(223)
			3200*g*, 5 min	
			20,000*g,* 25 min	
			80,000*g*, 1.5 h	
U87	225 mM mannitol, 75 mM sucrose, mM EDTA, 30 mM Tris-HCl, protease inhibitor cocktail	motor-driven homogenizer with an overhead stirrer (2000 rpm); tissue grinder set with a smooth PTFE pestle (Wheaton Potter-Elvehjem)	differential centrifugation	(224)
			1000*g*, 5 min	
			7000*g*, 10 min	
			100,000*g*, 30 min	
			followed with discontinuous sucrose gradient ultracentrifugation	
			2 M, 1.6 M, 1.2 M sucrose buffer	
			cell membrane: 1 M density	
U-251 MG	distilled water	high-pressure homogenizer (6000 psi/412.5 bar, 10 passages)	differential centrifugation	(67)
			10,000*g*, 10 min	
			60,000*g*, 90 min	
LNCaP-AI	Mem-PERPlus membrane protein extraction kit	stirring for 10 min	700*g,* 5 min	(225)
			16,000*g*, 15 min	
H22	18 mM Tris-HCl, 9 mM KCl, 1.5 mM MgCl_2_, protease inhibitor cocktail	Freeze–thawing, bath ultrasonic cleaner (42 kHz, 100 W)	differential centrifugation	(226)
			3000*g,* 6 min	
			20,000*g,* 25 min	
			120,000*g,* 1 h	
Stem Cells				
neural stem cells	10 mM Tris, 10 mM MgCl_2_, protease inhibitor cocktail	freeze–thawing	differential centrifugation	(188)
			7000*g,* 10 min	
			13,000*g,* 60 min	
human adipose-derived stem cells	10 mM Tris, 10 mM MgCl_2_, protease inhibitor cocktail	IKA T10 basic homogenizer (22,000 rpm for 1 min)	6000 *g,* 15 min	(227)
Miscellaneous				
MIN6	20 mM Tris-HCl, 10 mM KCl, 2 mM MgCl_2_, protease inhibitor cocktail	Dounce homogenizer, tight-fitting pestle (20 passes)	differential centrifugation	(96)
			20,000*g*, 20 min	
			100,000*g*, 45 min	
C6/36	20 mM Tris HCl, 10 mM KCl, 2 mM MgCl_2_, protease inhibitor cocktail	Dounce homogenizer, tight-fitting pestle (20 passes)	differential centrifugation	(228)
			3200*g*, 5 min	
			20,000*g,* 25 min	
			100,000*g*, 35 min	

a Abbreviations/cell lines: min, minutes, h, hour; BSA, bovine serum albumin; EDTA, ethylenediaminetetraacetic acid; EGTA, ethylene glycol-bis(β-aminoethyl ether)-*N*,*N*,*N*′,*N*′-tetraacetic acid; Tris-HCl, tris(hydroxymethyl) aminomethane (THAM) hydrochloride; MgCl_2_, magnesium chloride; KCl, potassium chloride; PMSF, phenylmethylsulfonyl fluoride; HEPES, 4-(2-hydroxyethyl)-1-piperazineethanesulfonic acid; DTT, dithiothreitol; DNase, deoxyribonuclease; RNase, ribonuclease; NK-92, human natural killer cell line; RAW 264.7, murine macrophage cell line; J774, murine macrophage cell line; THP-1, human acute monocytic leukemia cell line; HeLa, human cervical cancer cell line; MCF-7, human breast cancer cell line; 4T1, human breast cancer cell line; B16-F10, murine melanoma cell line; LL/2, murine lewis lung carcinoma cell line; CMT64.OVA, lung carcinoma cell line expressed ovalbumin; MB49, murine bladder carcinoma cell line; A549, human nonsmall cell lung cancer cell line; SKOV-3, ovarian cancer cell line; CAL27, human squamous cancer cell line; H22, murine hepatocarcinoma cell line; K562, human myelogenous leukemia cell line; MDA-MB-435, human breast carcinoma cell line; DU145, human prostate cancer cell line; CAL27, human squamous cancer cell line; U-251 MG, glioblastoma multiforme cell line; HCT116, human colorectal cancer cell line; U87, human primary glioblastoma cell line*;* MIN6, pancreatic β-cell line; C6/36, *Aedes albopictus* mosquito medium host cell line; LNCaP-AI, prostate cancer cell line.

### Nucleus-Free Cells

RBCs and platelets do not contain nuclei, making their membrane extraction process relatively simple. These cells are isolated first from whole blood using appropriate methodologies. For RBCs, hypotonic treatment easily ruptures the cells followed by centrifugation to collect a pink RBC membrane/ghost pallet. Multiple cycles of centrifugation remove hemoglobin impurities from the pallet.^62,210,211^ For platelets, it is common to use multiple freeze–thaw cycles to damage their cell membrane by breakage of ice crystals to remove the cytosol followed by centrifugations to obtain the cell membrane.^93,212^ According to one report, the obtained platelet vesicles were subjected to a discontinuous sucrose gradient (5%, 40%, 55%) step to remove any free proteins, intact platelets, and high-density granules to collect pure platelet vesicles from the interface of 5% and 40% sucrose gradient.^213^

Bacteria are interesting exceptions in this nucleus-free cell category. Besides containing peptidoglycans in addition to the cell membrane, their cell membrane extraction process can be laborious.^189,190^ Therefore, they undergo ultrafiltration to separate their membrane as OMV without a cell lysis step. The reported protocols for isolating OMVs and EVs from Gram-negative (*E. coli, K. pneumonia*) and Gram-positive (*S. aureus*) bacteria, respectively, are quite similar. In the first step, the bacterial cultures were centrifuged, and the supernatant was collected. The supernatant was further vacuum filtered through a micron filter and concentrated using ultrafiltration. Finally, the obtained filtrate was subjected to ultracentrifugation to get OMV pellets or EV pellets.^94,192,193^

Some groups reported further purification of these OMVs or EVs with some modifications. For example, after the first ultrafiltration step, the concentrate was reprecipitated overnight using ammonium sulfate (4 °C) and ultracentrifuged to get *E. coli* OMVs.^194,195^ OMVs resuspended in PBS were further purified using sucrose gradient (1 mL each of 2.5, 1.6, and 0.6 M sucrose), separated by ultracentrifugation. In another report, after ultrafiltration and ultracentrifugation steps, the obtained *S. aureus* EVs pellet was resuspended in 50% Optiprep/HEPES (2.2 mL).^190^ The suspension was applied to the bottom of a step-density gradient (2.0 mL of 40% and 0.8 mL of 10% Optiprep in 10 mM HEPES, supplemented with 150 mM NaCl, pH 7.0), and obtained the pure *S. aureus* EVs floating at 1.16–1.20 g/mL.

### Nucleus-Containing Cells

For nucleus-containing cells, cell membrane isolation and purification are slightly more tedious than that with nucleus-free cells. Examples include immune cells (macrophages/monocytes, neutrophils, NK cells, T cells), cancer stem cells, fibroblasts, and β-cells. These cells can either be obtained from established cell lines (like human breast cancer cell lines (MCF-7, 4T1), mouse macrophage cell line (J447), human NK cell line (NK-92), *etc.*), or isolated from tissues or blood (neutrophils, cancer cells, T cells, NK cells, stem cells).

On average, 200–300 million cells are required for cell membrane isolation to assemble a CMC mimic.^63,67^ These cells are ruptured using the hypotonic treatment and physical disruption techniques, resulting in a mixture containing pure cell membrane, intact cells, and high-density granules. Differential centrifugation or discontinuous sucrose gradient ultrafiltration of the mixture finally isolates the cell membrane. These methods are described in detail below.

Differential centrifugation method: This method is the one most commonly used for isolating cell membranes.^55,130,150^ It works by a stepwise increase in the centrifugation speed. The lower *g* at the beginning of the process removes heavy particles like a nucleus. A gradual increase in *g* removes other particles like mitochondria. Finally, very high *g* is used to pellet down the cell membrane, as it is lighter in weight. For example, the commonly reported centrifugation speeds for isolating cell membrane are 800 *g* (4 °C, 10 min), followed by 10,000 *g* (4 °C, 30 min), and finally 100,000 *g* (4 °C, 60 min) to isolate pure cell membrane.^175,214^

Discontinuous sucrose gradient ultracentrifugation method: In this method, sucrose concentration increases discretely from top to bottom, aiding density-based separation of particles in the solution. The particles move across the density gradient stopping in a region where their density matches that of the medium. For example, this method was used to demonstrate the isolation of leukocyte cell membrane using 55%, 40%, and 30% (w/v) sucrose gradients in a physiological saline solution.^63^ The cell membrane was collected from a 30/40% interface with minimal/no nuclear and mitochondrial contamination. A similar approach has been preferred to isolate several cell membranes using this method.^63,98,102^

During the membrane isolation there can be a loss of functional components like transmembrane proteins/receptors or structural components like cholesterol from the membrane. Cholesterol is mainly responsible for maintaining the rigidity of the cell membrane.^215,216^ Such loss may result in a decrease in the mechanical stability of the membrane. Therefore, to reduce protein loss and maintain membrane stability, hypotonic buffers with divalent ions (like MgCl_2_) or the addition of cholesterol can be useful. These stabilize the membrane skeleton by specifically binding to the junction complex and other membrane proteins like tropomyosin, *etc*.^217,218^ Additionally, mild lysis buffers, gentle rupturing techniques, the right pH, and ice-cold conditions must be used for membrane isolation to prevent the degradation of the transmembrane proteins and receptors.

### Intracellular Organelle

Cell membrane isolation of intracellular organelle requires additional steps, unlike nucleus-containing cells. Before cell membrane isolation, it is essential to first isolate the desired organelle from nucleus-containing cells in their pure form. Isolating organelles from cells is a three-step process: hypotonic treatment, physical disruption, and ultracentrifugation. The final step is carried out in a sucrose density gradient to get the purified organelle in a specific sucrose band. The process is repeated with the purified organelle to extract the pure cell membrane. The mitochondrial outer membrane was isolated from the mouse liver using a similar protocol^91^ (Table 2).

## Choice of Template Based on Its Properties

The template is a central component of a CMC mimic that provides a structural basis during its assembly. Inherent properties of templates extend the application of CMC mimics for diagnosis, drug delivery, and disease suppression/treatment.

There are two major categories of templates (spherical or nonspherical): organic and inorganic. Poly(lactic-*co*-glycolic acid) (PLGA), gelatin, and liposomes are examples of organic templates. Mesoporous silica, gold and iron oxide (Fe_3_O_4_), upconversion nanoparticles (UCNPs), persistent luminescent nanoparticles (PLNPs), and metal–organic frameworks (MOFs) are examples of inorganic templates. Organic templates offer features like biocompatibility, biodegradability, and nontoxicity and are often straightforward choices.^229^ In comparison, inorganic templates display additional features like magnetic, optical, and electrical properties that determine their selection in a CMC mimic.^230^

**Figure 3:**
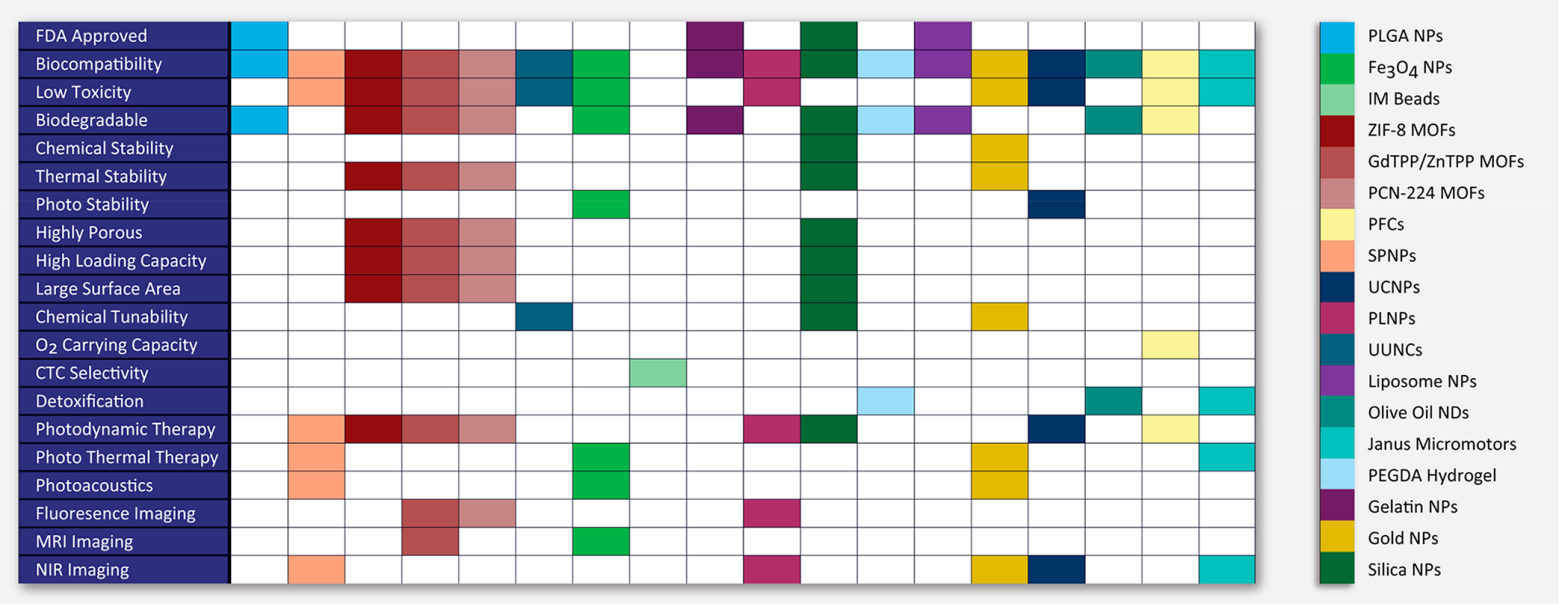
The schematic highlights specific physiochemical and biological properties ingrained in a CMC mimics by selecting an appropriate template. The template is one of the components that provide additional value in CMC mimics. The schematic provides a summary of all the possible properties that help in the choice of the reported template while designing CMC mimics. Abbreviations: FDA, Food and Drug Administration; O_2_, oxygen; Fe_3_O_4_, iron oxide; CTC, circulatory tumor cells; MR, magnetic resonance; NIR, near-infrared; PLGA, polylactic-*co*-glycolic acid; IM, immunomagnetic; ZIF, zeolitic imidazolate; MOFs, metal–organic frameworks; GdTPP, gadolinium porphyrin; ZnTPP, zinc porphyrin; PCN-224, porphyrin (TCPP)-based Zr_6_ cluster; PFCs, perfluorocarbons; NPs, nanoparticles; MPs, microparticles; NDs, nanodroplets; SP, semiconducting polymer; UC, upconversion; PL, persistent luminescent; UUNCs, ultrasmall unimolecular nanoclusters; PEGDA: poly(ethylene glycol) diacrylate.

In this section, we have provided the general overview of the templates categorized based on their properties in the context of CMC mimics like Food and Drug Administration (FDA) approval, biocompatibility, biodegradability, and low toxicity to understand the clinical translation perspective, phototherapy for cancer suppression/treatment, bioimaging for disease diagnosis, and detoxification for enhancing the removal/absorption of toxins (summarized in Figure 3).

### FDA-Approved, Biocompatible, Biodegradable, Low Toxicity Templates

CMC mimics are biocompatible, as the cell membrane protects every template from the external microenvironment. For clinical translation, it is also vital to consider template biodegradability and biocompatibility. Byproducts after the biodegradation and their interaction with the human body also determine their toxicity.^231^ Renal clearance helps to evade undesirable side effects.^231^ FDA-approved templates are considered the safest, nontoxic, or nonhazardous to the human body in every aspect. These properties help to protect the healthy cells in the body and avoid any unwanted immune response.

Most organic templates are generally thought to be safer than inorganic templates and were, therefore, entered easily into clinical trials.^232,233^ Examples of organic templates used in CMC mimics are PLGA, gelatin, and liposomes that are FDA approved, biocompatible, and biodegradable in nature. In 2011, the possibility of designing these mimicking systems was demonstrated using a PLGA nanoparticle as a template.^62^ PLGA is a versatile synthetic polymer that molds into both nano- and micro-sized particles. It is the most common template used for several cell membrane coatings like RBC,^62,114,211,234^ platelets,^87,93,121,123^ cancer cells,^65,92,235^ neutrophils,^55,66^ cardiac stem cells,^187^ macrophages,^130^ NK-92 cells,^67^ dendritic cells,^106^ and so on. Gelatin is a natural polypeptide used in cosmetics, pharmaceuticals, the food industry, and in the assembly of CMC mimics.^236^ For designing CMC mimics, several cell membranes used for coating on gelatin templates are RBC,^64^ stem cell,^186^ T cell,^237^ mosquito medium host *Aedes albopictus* (C6/36) cell,^228^ and patient-derived tumor cells.^105^ Liposomes are spherical vesicles having at least one lipid bilayer. Liposomes have been used for coating macrophages,^129^ RBCs,^238^ and cancer cell membranes.^175^ As reported in the literature, liposomes can also easily fuse with cell membrane vesicles like RBC^239^ and NK-92 cells^102^ for designing CMC mimics. Perfluorocarbons (PFCs) are another example of a regulatory-approved template. In 1989, PFCs (Fuosol-DA) were approved in the US, Japan, and Europe for clinical use but were taken off from the market after 5 years due to difficulties in their storage-related issues.^240^ Nevertheless, PFCs are biocompatible and biodegradable and have a high oxygen-carrying capacity. Many PFCs have a capacity for oxygen dissolution that is nearly 20 times that of water. They can, moreover, be easily fabricated at the nanoscale for oxygen delivery even to the smallest capillaries.^241^ Therefore, several CMC mimics reported using PFC can supply oxygen at the tumor sites to relieve hypoxic conditions.^242,243^

Most of the inorganic templates are biocompatible, but toxicity depends on the metal used for their synthesis and its degradation in the cell. Among inorganic templates, mesoporous silica is considered the safest (approved by FDA) and is biocompatible and biodegradable.^244^ It degrades into nontoxic silicic acid (water-soluble).^245^ It has been a popular template for many years in research due to its high porosity, large surface area, and high drug/photosensitizer loading capacity.^246^ CMC mimics reported with spherical silica nanoparticles used several cell membranes from RBCs,^61^ cancer cells,^247^ and macrophages.^247^ Other templates like liposome-PEG,^175^ UCNPs,^185^ and PLNPs^68^ were used in combination with silica to increase their drug/photosensitizer loading capacity. Mesoporous silica nanoparticles are tunable to different sizes and shapes.^248,249^ According to the reports, rod-shaped silica nanoparticles can enhance antimicrobial properties^250^ and regulate the endogenous reactive oxygen species for oxidative therapy.^251^ These tunable properties coupled with CMC mimics could offer potential therapeutic benefits if explored further. Silica templates can also have several desired surface functionalities post-chemical modifications.^252^ For example, positively charged 3-aminopropyl triethoxysilane (APTES) was used to modify the surface charge of silica microparticles to coat a negatively charged leukocyte membrane^115^ and platelet membrane for CTC detections.^213^ For Fe_3_O_4_ nanoparticles, iron ions are its biodegradation byproducts and are mostly nontoxic.^231^ Several CMC mimics reported using Fe_3_O_4_ templates used cell membranes like macrophage,^131^ MSCs,^95^ and HeLa cells.^173^ Similarly, MOFs are well-defined 3D architectures formed by the complexation between organic ligands and inorganic metal ions.^253^ These are biocompatible, and their toxicity depends on the nature of the metal and organic linker used. For example, zinc-based MOFs (zeolitic imidazolate (ZIF-8)) release Zn^2+^ ions post-degradation, an endogenous element that causes a less harmful effect on the human body if present in a low amount.^254^ MOFs of porphyrin (TPP)-based Gd/Zn nanocomposites release gadolinium (Gd^3+^) and zinc (Zn^2+^) ions post-degradation. Gd^3+^ can cause a toxic effect in the abnormal functioning of kidneys and cross the blood–brain barrier to accumulate in the brain.^255^ Several CMC mimics reported using cancer cell membrane-coated MOFs for homologous targeting.^219,256−258^ MOFs also have high porosity, large surface area, and high photosensitizer loading capacity^256−258^ due to their structural arrangement. Gold particles are another commonly used biocompatible inorganic template because of their inert nature. However, they are not biodegradable and may be cause for concern.^259^ To overcome these issues, the use of nano or ultrasmall templates to facilitate rapid renal clearance is preferred.^231,260^ Gold particles are tunable to different shapes: nanoparticles, nanocages, nanorods, and nanoshells, and all are used as templates for fabricating CMC mimics. Examples of these are gold nanocages with RBC membrane coating^56^ and H22 liver cancer cell membrane coating,^226^ nanorods with RBC membrane^261^ and platelets membrane coating,^262^ and nanoshells with macrophage membrane coating^139^ for a specific application.

### Phototherapy

Phototherapy is a noninvasive and effective cancer treatment. It includes photothermal therapy (PTT) and photodynamic therapy (PDT).^263^ With the right choice of template, these photothermal or photodynamic properties can be explored with CMC mimics. PTT involves the photo absorbing agents to generate heat under near-infrared region (NIR) laser irradiation to kill cancer cells thermally and is less harmful to other cells or tissues.^264^ Gold templates have a large NIR absorption cross-section and tunable localized surface plasmon resonance (LSPR) band in the NIR region.^226^ This makes them most suitable to incorporate in CMC mimics for PTT.^221,226^ Similarly, magnetic templates like Fe_3_O_4_ are also good alternatives for their use in CMC mimics for PTT. Fe_3_O_4_ templates are efficient in photothermal conversion and are outstanding options for hyperthermia treatment.^265^ Fe_3_O_4_ nanoclusters showed a significant increase in NIR absorption,^265^ in contrast to their nanoparticles.

PDT involves reactive oxygen species (ROS) generation with photosensitizers under the light of a specific wavelength for oxidation and killing cancer cells. Mainly ROS are like singlet oxygen (^1^O_2_), superoxide anion radical (O^2-•^), or hydroxyl radical (^•^OH).^266^ Some combinations of photosensitizers and templates used together in CMC mimics are chlorin e6 (Ce6) in hollow mesoporous silica,^267^ merocyanine 540 (MC540) in UCNPs,^268^ zinc phthalocyanine (ZnPC) and MC540 in mesoporous silica encapsulated UCNPs,^185^ 5,10,15,20-tetraphenylchlorin (TPC) in (ROS)-responsive paclitaxel (PTX) dimer (PTX2-TK),^79^ and silicon phthalocyanine in PLPNs.^72^ PFCs used in combination with photosensitizers provide an adequate oxygen supply to accelerate the generation of reactive singlet oxygen (^1^O_2_) and enhance PDT therapy.^269^ In porphyrin-based MOFs,^168,219,257^ porphyrin acts as a photosensitizer due to its ability to readily absorb visible light and improve overall ROS generation efficiency.^270^ The template used in CMC mimics for both PDT and PTT is a semiconducting polymer (SP) nanoparticles-poly(cyclopentadithiophenealt-benzothiadiazole) (PCPDTBT)). SP nanoparticles are known for their excellent optical properties and high NIR absorbing capacity and can generate signet oxygen and heat.^104^ Verteporfin is a photodynamic agent approved by the US FDA for eliminating abnormal blood vessels in the eyes.^271^ Recently, platelet membrane-coated verteporfin loaded PLGA nanoparticles reduced skin damage in PDT in combination with solar radiation.^121^ Indocyanine green (ICG) is an FDA-approved photosensitizer and photothermal agent for template encapsulation.^174,209,269,272^

### Bioimaging

Bioimaging technology has significantly enhanced the ability to diagnose, treat, and prevent diseases by enabling early detection. It helps in imaging inside the animal and human body. Bioimaging includes magnetic resonance imaging (MRI), near-infrared (NIR) imaging, and fluorescence (FL) imaging.

Fe_3_O_4_ nanoparticles are the most commonly used as negative (T_2_) contrast agents for MRI in CMC mimics.^57,265,273^ Currently, standard probes used in MRI scans are gadolinium (Gd^3+^)-based compounds. These are positive (T_1_) contrast agents in MRI and also have been a preferred choice in the clinic for their better image resolution and easy detection, tunable magnetic properties, and higher colloidal stability.^274^ But these agents also limit their use in patients with renal imparitment and have been reported to cross blood–brain barriers to accumulate in the brain.^255^ Some examples of Gd^3+^-based templates used in CMC mimics are PLGA-Gd-lipid^67,71^ and MOFs like porphyrin (TPP)-based Gd/Zn nanocomposites.^219^ Manganese (Mn^2+^) ions can also be a potential alternative to gadolinium as positive (T_1_) MRI contrast agents.^275^ Since Mn^2+^ is one of the essential elements in the human body, its intake in small amounts does not produce toxic effects.^274^ Recently, CMC mimics designed using porphyrin (TCPP)-based Zr^4+^ clusters MOFs-coated with MnO_2_ nanosheets converted MnO_2_ into Mn^2+^ because of the generation of H_2_O_2_ in the system used for MRI.^256^ Porphyrin-based MOFs can absorb the energy produced by the excitation of light and generates fluorescence for imaging.^270^ Gold nanoparticles, PLNPs, UCNPs, and semiconducting polymer (SP) nanoparticles are examples of templates in CMC mimics used for NIR imaging. Gold templates have a large NIR absorption cross-section and a tunable LSPR band in the NIR region, providing greater penetration depth in the imaging.^226,276^ PLNPs have a long-lasting near-infrared afterglow and avoid tissue autofluorescence from *in situ* excitation.^68,72^ SP nanoparticles have a high NIR absorption capacity.^104^ UCNPs have significant light penetration depth, narrow emission peaks, no background fluorescence, and exceptional photostability.^132,277^ Indocyanine green (ICG) is best known for NIR fluorescence imaging^65^ along with phototherapy.

### Detoxification

Detoxification removes infections caused by pathogens. The RBC membrane alone^278^ or in combination with platelet membrane^59^ has toxin absorbing capabilities. There are also some templates/devices used in the CMC mimics to enhance the detoxification process differently. These include olive oil nanodroplets, Janus micromotors, redox-responsive hydrogels, and a 3D bioprinted nanoparticle-hydrogel hybrid device.

RBC membrane wrapped olive oil nanodroplets were used to form biomimetic oil nanosponges.^77^ In these nanosponges, the olive oil core soaked nonspecific toxicants through the physical partition, and RBC absorbed and neutralized toxicants through biological binding. They also found greater detoxification than that obtained with PLGA-RBC nanosponges. RBC membrane-coated antibiotic-loaded redox-responsive hydrogels (RBC-nanogels) were reported to absorb and neutralize the pore-forming toxins in the extracellular environment.^114^ This facilitated their intracellular uptake into the bacteria. Once entered within the bacteria, the cross-linked hydrogel cleaved to release the antibiotics to inhibit the bacterial growth. These redox-responsive hydrogels were more effective in inhibiting bacterial growth than the free antibiotics and nonresponsive hydrogels. Further, RBC membrane-coated Janus micromotors were used to improve the speed of absorption and neutralization of both nerve agent stimulants and biological protein toxins.^76^ The water-driven mimicking systems were designed by integrating RBC membranes, gold nanoparticles, and alginate (ALG) onto the exposed surface areas of magnesium (Mg) microparticles partially embedded in parafilm. This partial embedding leads to a small hole in the Mg particles. Hydrogen bubbles produced by the spontaneous redox reaction between Mg and water provided the guided propulsion without any external fuel. The 3D bioprinted nanoparticle-hydrogel hybrid device was designed with multiple inner channels for encapsulating many RBC nanoparticles.^201^ Many RBC nanoparticles in one device enhanced the detoxification process while at the same time absorbing various nonspecific toxins flowing through the channel.

## Assembly of Cell Membrane-Coated Mimics

The most crucial step in designing a CMC mimic is the assembly of extracted cell membranes from the cell of interest with the template of choice that can incorporate its physiochemical properties to the CMC mimics. The isolated cell membrane may either be in the form of fragments or of vesicles. Before coating, it may be necessary to include an additional extrusion^56,69,92^ or sonication^55,93,279,280^ steps to form cell membrane vesicles (Figure 2). This section describes commonly employed CMC assembly techniques. We have also highlighted other less explored assembly techniques such as microfluidic electroporation, *in situ* polymerization, and graphene nanoplatform-mediated cell membrane coating. Additionally, we will emphasize the scope and challenges of assembly processes, manufacturing difficulties (reproducibility and scale-up), and limitations for clinical translation.

### Extrusion

Producing uniformly sized particles by pushing material through a porous membrane of the desired cross-section is called extrusion.^281^ The formation of a wide range of nanomaterials like nanoparticles, liposomes, nanotubes, nanofibers, and emulsions use of extrusion technique is preferred. The commonly used membrane extrusion strategies are vesicle extrusion (for liposomes),^282^ membrane emulsification (for emulsions),^283,284^ precipitation extrusion (for nanofibers and nanoparticles),^285^ and biological membrane extrusion (for CMC mimics).^62,286^

For fabricating CMC mimics, a solution of the cell membrane vesicles and the template repeatedly passes through a porous polycarbonate membrane in a mini extruder. The mechanical force applied during the process disrupts the membrane structure and helps it to wrap around the template. In 2011, extrusion was reported for uniform coating of an RBC cell membrane onto a PLGA nanoparticle template through 400 and 100 nm polycarbonate porous membranes.^62^ Since then, several groups have reported this technique for assembling CMC mimics using different pore sizes of polycarbonate membranes, cells, and template types.^56,69,130,132,186,235^ After repeated extrusion, centrifugation separates the left/unbound cell membrane vesicles from the mixture. The main limitation of this technique is the loss of sample due to the accumulation of the material on the porous membrane, leading to difficulty in large-scale production.

### Sonication

Sonication is the process of applying sound energy to disperse the particles in the liquid using an ultrasonic bath or probe sonicator. In this technique, both the cell membrane and the template are co-incubated, followed by sonication in ice-cold conditions for few minutes to fabricate CMC mimics. Sonication disrupts the cell membrane layer, and the noncovalent interactions between the template and the cell membrane facilitate their assembly. Several groups have reported this technique for CMC mimic assembly using different cell and template types, for example, RBC membrane coating onto cross-linked 2-hydroxyethyl acrylate (HEA) hydrogel microparticles;^287^ cardiac stem cell membrane onto PLGA microparticles;^187^ and stem cell,^227^ platelet,^93^ neutrophil membranes^55^ onto PLGA nanoparticles; and a hybrid of RBC and platelet membrane onto gold nanowires.^196^ After the sonication, centrifugation of the mixture separates the left/unbound cell membrane vesicles. Sonication, unlike the extrusion technique, avoids the loss of material during the coating process. It requires optimization of parameters like inputs of power, frequency, and time to avoid sample damage or denaturation of protein due to heat energy. However, the resulting particles may vary in size and uniformity of coating.^187,227^ This technique might also not be appropriate for soft/some templates, as it might affect their size and stability.^288,289^

### *In Situ* Polymerization

*In situ* polymerization is a technique of preparing nanocomposites. It consists of polymeric molecules bound to nanoparticles^290^ (like carbon nanotubes, graphene oxide, *etc.*) or to biomolecules^291^ (like DNA, RNA, or proteins, *etc*.) in a reaction polymerization mixture to form linear conjugates or nanocapsules. The reaction mixture consists of a monomer, initiator, and a cross-linker, exposed to a source of heat or radiation to initiate the polymerization mechanism.

In 2015, this technique was reported using RBC membrane-derived vesicles as a nanoreactor to synthesize polymeric cores *via in situ* polymerization to prepare cell membrane-coated hydrogel nanoparticles.^114,292^ Membrane vesicles were prepared by extruding a mixture containing RBC ghosts, monomer (acrylamide), cross-linker (*N*,*N*′-methylene bisacrylamide), and an initiator (lithium phenyl-2,4,6-trimethylbenzolyphosphinate) through a polycarbonate membrane filter. A PEG-modified 2,2,6,6-tetramethylpiperidin-1-yl)oxyl (TEMPO) inhibitor was added to this solution to prevent cross-linking of monomers on the outside of the cell membrane vesicles. This inhibitor selectively promotes *in situ* cross-linking, protects outer cell proteins from denaturing, and inhibits nonspecific interactions and leaking of inner monomers across the cell membrane. Upon UV exposure for 5 min, monomers inside the cell membrane selectively polymerized to form a stable template at room temperature. This process is opposite to the traditional coating methods. It has the potential to be extended for other cross-linking mechanisms and materials and for templates-cell membrane combinations that are not currently feasible due to their unfavorable surface properties. However, preparing cell membrane vesicles using extrusion technique can lead to sample loss during large-scale production.

### Microfluidic Electroporation

Electroporation is a high-throughput technique of incorporating nanoparticles within cells.^293,294^ In this technique, cells subjected to rapid high-voltage electric field pulses create temporary hydrophilic pores within the cell membrane.

In 2017, the microfluidic-assisted fabrication of CMC mimics was demonstrated using electroporation.^57^ An electroporation setup was integrated with a microfluidic chip with an S-shaped channel to facilitate efficient mixing of RBC vesicles and nanoparticles, fed through a Y-shaped polydimethylsiloxane microchannel. During electroporation, the pores formed with the cell membrane allow passive transport of nanoparticles within the RBC vesicles and fabrication of uniformly coated RBC-Fe_3_O_4_ nanoparticles with improved colloidal stability, uniform size, and *in vivo* efficacy. The advantages of this technique are in the autologous extraction of RBCs, allowing for personalized diagnosis and therapy. Scalability and storing capacity of this technique promote its feasibility for industry translation.

### Graphene Nanoplatform-Mediated Cell Membrane Coating

In 2019, a single-step methodology for extraction and assembly of the leukocyte cell membrane was reported.^295^ The design aimed to increase the antileukocyte targeting ability of CMC mimics using a leukocyte cell membrane. The selective ability of graphene nanosheets to extract phospholipids from the cells vigorously was the innovative aspect of this CMC mimic. Initially, negatively charged Fe_3_O_4_ magnetic nanoparticles were modified with graphene and prepared by the layer-by-layer technique. A positively charged polyethylenimine (PEI) facilitated the immobilization of negatively charged graphene nanosheets onto Fe_3_O_4_ nanoparticles. The CMC mimics were assembled in a quick single-step process by co-incubating graphene-modified nanoparticles with leukocytes in serum-free media. High phospholipid content on the surface of CMC mimics helped to immobilize lipids for antibody conjugation to target epithelial cell adhesion molecule (EpCAM)-positive CTCs, for example, MCF-7 (the human breast cancer cell line) and HepG2 (human hepatocellular cancer cell line). They also demonstrated CMC mimics’ selectivity with a very high antileukocyte targeting efficacy when tested in synthetic samples (blood mixed with green fluorescent protein (GFP)-MCF-7 cells). The advantage of this protocol is the selective extraction and immobilization of phospholipids from different cell types^296^ and the efficient separation and proliferation of the captured CTCs for several passages. Further, it is possible to use these CTCs to design a biomimetic system with homotypic target abilities.

## Characterization of CMC Mimics

### Physiochemical Characterization

**Figure 4:**
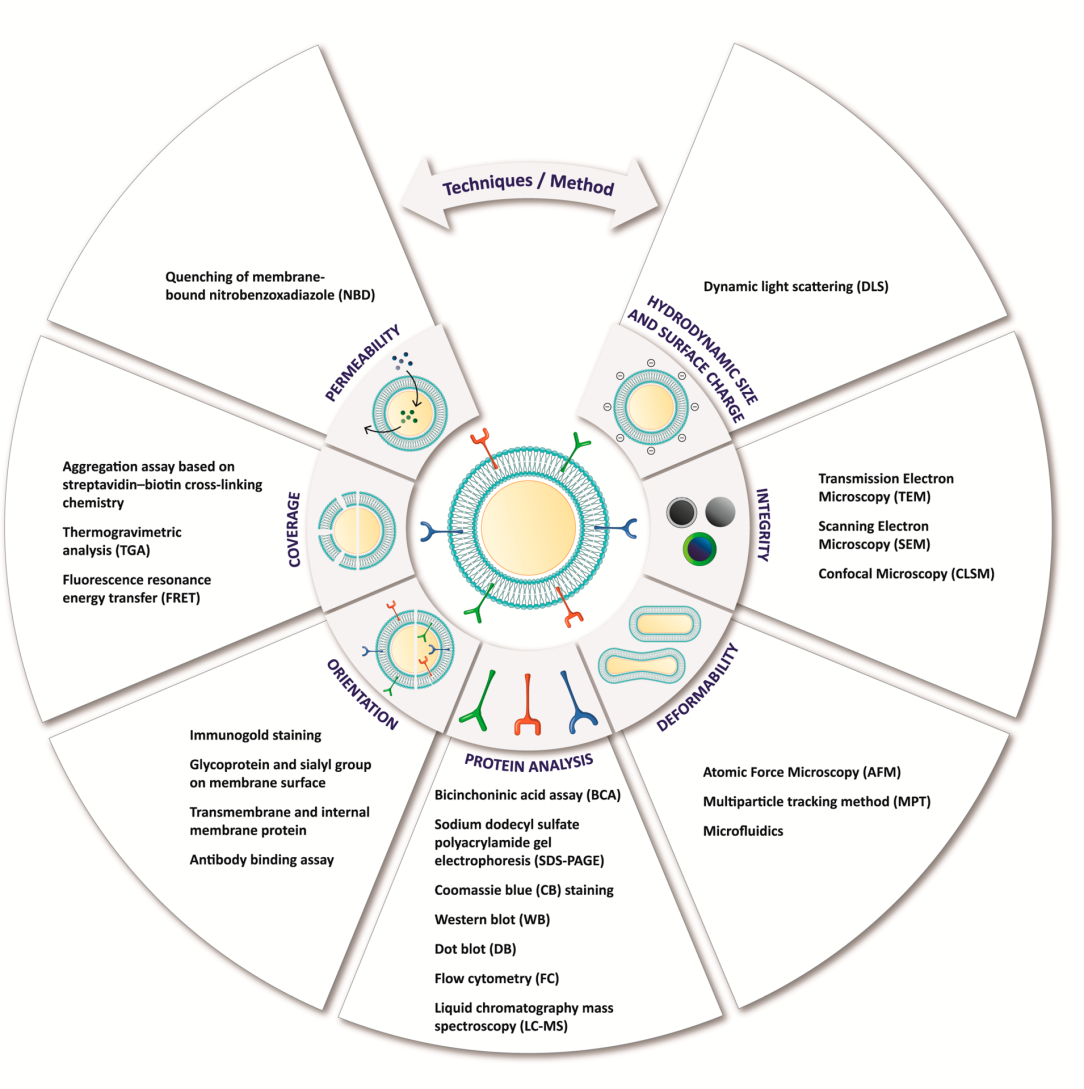
The schematic illustrates the qualitative and quantitative physicochemical and biological properties of CMC mimics that validate their formation. Some essential parameters need to be considered while designing CMC mimics like surface charge, the thickness of cell membrane-coated onto a template, elasticity, protein quantification and identification of right orientation, amount and area of cell membrane covered onto a template, and permeability of the mimics for the diffusion process. This helps in confirmation and visualization of the cell membrane with right-side-out in CMC mimics. The schematics also list the methods and instruments used for characterizing specific physicochemical and biological properties of CMC mimics.

After the fabrication of a CMC mimic, it is essential to analyze its structural features involving the cell membrane and template interface to enhance its colloidal stability. The incomplete/unstable membrane may lead to template exposure and impair the effectiveness of the CMC mimics. Therefore, it is critical to perform qualitative and quantitative evaluations of their structural integrity. The quantitative determination of the number of templates coated with the cell membrane remains unexplored even though this is an important parameter for its clinical translation. In this section, we collate all reported physiological techniques to quantify and visualize thickness, uniformity, stability of the cell membranes, and deformable and permeability properties of CMC mimics post-assembly (Figure 4).

#### Size and Surface Charge

**Figure 5:**
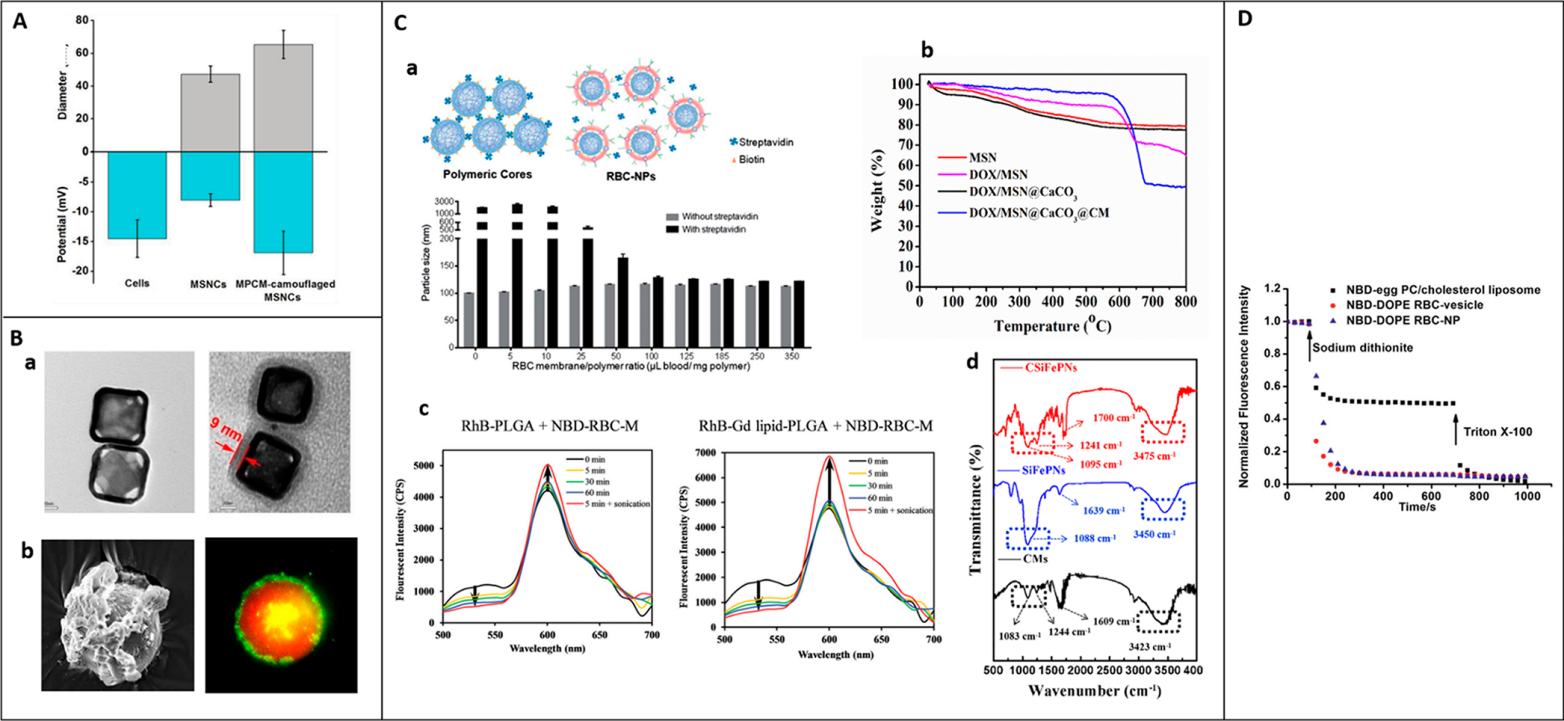
Surface charge, hydrodynamic diameter, coverage, and permeability. (A) Size and surface charge of mesoporous silica nanocapsules (MSNCs) and macrophage cell membrane (MPCM)-coated MSNCs. Reprinted with permission from ref (69). Copyright 2015 John Wiley and Sons. (B) Structural integrity: (a) TEM image of gold nanocages (left) and H22 liver cancer cell membrane-coated gold nanocages (right), scale bar = 20 nm. Reprinted with permission under a Creative Commons CC BY-NC License from ref (226). Copyright 2018 Ivyspring International Publisher. (b) Cardiac stem cell membrane fragments-coated onto PLGA microparticles, SEM (left), confocal microscopic images (right) microparticles (texas red), cell membrane (DiO, green), scale bar = 20 μm. Reprinted with permission under a Creative Commons CC-BY License from ref (187). Copyright 2017 Springer Nature. (C) Coverage: (a) Aggregation assay based on streptavidin-biotin cross-linking chemistry, size change of RBC-NPs (RBC-coated PLGA nanoparticles) due to streptavidin-biotin cross-linking at various RBC membrane-to-polymer ratios. Reprinted with permission under a Creative Commons CC-BY License from ref (300). Copyright 2014 Royal Society of Chemistry. (b) TGA curves of MSN, doxorubicin-loaded MSN (DOX/MSN), DOX loaded MSN with calcium carbonate interlayer (DOX/MSN@CaCO_3_), prostate cancer cell membrane-coated DOX/MSN@CaCO_3_ (DOX/MSN@CaCO_3_@CM). Reprinted with permission from ref (225). Copyright 2019 Elsevier. (c) FRET study to demonstrate the insertion of RBC-M onto the gadolinium inserted poly(lactic-*co*-glycolic acid) nanoparticles (Gd-PLGA) and bare PLGA. Reprinted with permission from ref (71). Copyright 2020 Royal Society of Chemistry. (d) FT-IR spectra of CMs (MDA-MB-231 cell membranes), SiFePNs (MSNs containing superparamagnetic ferroferric oxide), and CSiFePN (cell membrane coated mimics). Reprinted with permission under a Creative Commons CC-BY License from ref (247). Copyright 2019 Springer Nature. (D) NBD fluorescence quenching to study and compare the permeability of RBC vesicles, RBC-NPs (RBC coated PLGA nanoparticle), liposome (egg PC/cholesterol). Reprinted with permission from ref (302). Copyright 2014 Royal Society of Chemistry.

Size and surface charge are two parameters monitored in real-time during the assembly of a CMC mimic. Size (hydrodynamic radius) and surface charge of CMC mimics can be measured using a dynamic light scattering (DLS) analyzer and zeta sizer (Figure 5A).

Post-assembly of CMC mimics, it is typical to note a negative surface charge close to that of the cell membrane and a few nanometers increase in their size, confirming the coating.^64,94,100,198,226,257^ Measuring size pre and post-CMC assembly by using DLS helps to determine the thickness of the cell membrane. However, the thickness of outer membrane coating can vary depending on the number of layers and their extent of fusion with the template.^63^ In this section, we have mentioned a few variations of the template and cell membrane thickness in different CMC mimics.

For example, a T cell membrane-coated PLGA system was reported with an observed size change from 88.3 ± 1.3 nm to 105.4 ± 4.4 nm (thickness ∼17.1 nm) and a surface charge of −29.5 ± 1.2 mV similar to that of the cell membrane.^159^ In T1 cell membrane-coated cerium oxide dotted CS, there was an increase in 20 nm size from 131.7 ± 5.2 nm to 152.8 ± 3.9 nm post-assembly with −26.1 ± 0.9 mV ζ potential after cell membrane coating.^297^ In monocyte membrane (U837)-coated PLGA systems, there was an increase of size by ∼20–40 nm with −16.5 mV ζ potential (PLGA: −8.3 mV; U837: −13.6 mV).^298^ In MDA-MB-231 cell membrane-coated mesoporous silica loaded with ferric oxide, an increase in average size from 164 to 220 nm (thickness ∼56 nm) with surface charge −20.88 ± 0.4 mV post-assembly was observed.^247^ In fact, in MCF-7 cell membrane-coated mesoporous silica PEG-liposomes, the size change from 74.07 ± 0.7 nm to 188.5 ± 3.3 nm (thickness ∼114 nm) with −23.8 ± 1.1 mV surface charge closer to that of the cell membrane was observed.^175^ In addition to using a DLS analyzer, the thickness and coverage of the cell membrane on each template can be visualized using microscopic techniques discussed in the next section.

#### Structure Integrity

Different microscopic techniques help to visualize the structural integrity of CMC mimics. The three microscopic techniques most often used to gain insight into the structural integrity and uniformity of assembled CMC mimics are cryo-transmission electron microscopy (cryo-TEM) or TEM, field-emission scanning electron microscopy (FESEM) or SEM, and confocal laser scanning microscopy (CLSM) (Figure 5B).

CMC mimics have a characteristic core–shell structure. This consists of a dense inner core of a template and a thin outer coating of a cell membrane. Due to differences in composition, there is a difference in electron density between these two layers. However, TEM imaging visualizes the structure containing a dark core and a light outer coating. The variation in the thickness of the cell membrane in different CMC mimics was also visualized in TEM analysis, same as observed in the above section. For example, in the leukocyte membrane-coated silica-APTES system, the outer layer thickness could reach 500 nm by increasing the membrane:particle ratio.^63^ For leukocyte cell membrane-coated Fe_3_O_4_-PEI-graphene-modified nanoparticles, variable thickness of the membrane around 11.78 and 16.94 nm was observed.^295^ In case of hybrid RBC and MCF-7 breast cancer cell membrane-coated melanin nanoparticles, an ∼9.1 nm-thick membrane was reported.^198^ Similarly, for 4T1 cancer cell membrane-coated MOFs^257^ and RBC membrane-coated PFCs nanoparticles,^269^ an ∼10 and 20 nm-think membrane was observed, respectively. Overall, TEM can provide a qualitative estimation of the membrane homogeneity around the template post-coating, mostly in the case of nanoscale CMC mimics.

SEM is another qualitative technique used to visualize the change in surface morphology/texture after the cell membrane coating and complete/incomplete coverage of cell membrane, predominantly in the case of microscale CMC mimics. For example, using SEM, the complete coverage of leukocyte membrane onto APTES-silica microparticles^63^ and incomplete coverage of cardia stem fragments onto PLGA microparticles were observed.^187^ In case of motor sponge designed using RBC membrane and gold nanowires of 400 nm diameter and 3 μm in length, no change in the concave end of the nanowire was observed after complete RBC membrane coating.^299^ Uniform RBC membrane coating onto Mg Janus motors was observed with spherical geometry of size 20 μm and a small circular opening of 2 μm.^76^

CLSM is a qualitative technique that provides an insight into the efficiency of the whole process of assembly by determining an estimation of the number of templates that are well coated. For CLSM, the cell membrane and the inner core are fluorescently tagged with different dyes to aid visualization. 3,3′-Dioctadecyloxacarbocyanine perchlorate (DiO),^187,211,219,235^ 1,1′-dioctadecyl-3,3,3′,3′-tetramethylindodicarbocyanine perchlorate (DiD),^75,175,185^ 1,1′-dioctadecyl-3,3,3′,3′-tetramethylindocarbocyanine perchlorate (DiI),^239^ fluorescein,^55^ rhodamine-DMPE (1,2-bis(dimethylphosphino)ethane),^62^ wheat germ agglutinin,^63^ rhodamine B (RhB),^299^ CellVue,^287^ and Cy5.5^60^ are examples of dyes exclusively used to label cell membranes.

#### Coverage

The coverage of cell membrane onto the template can be validated using an aggregation assay based on streptavidin-biotin cross-linking ability, Förster resonance energy transfer (FRET) analysis, thermogravimetric analysis (TGA), and Fourier transform infrared (FT-IR) spectroscopy (Figure 5C).

In 2014, the completeness of RBC membrane was demonstrated onto PLGA nanoparticles using the streptavidin-biotin cross-linking chemistry (aggregation assay).^300^ The RBC membrane-coated biotinylated PLGA nanoparticles were mixed with the streptavidin and monitored for a change in particle size. When the membrane coverage is low, exposed biotin on the PLGA surface binds to streptavidin and induces significant aggregation and size change. Therefore, the suitable membrane to polymeric ratio can be optimized when the aggregation or size changes upon the addition of streptavidin impede. Such an aggregation assay helps in determining the efficiency of membrane coating in CMC mimics.

TGA technique measures the percentage loss in weight of samples when heated.^301^ The TGA profiles can differ from material to material (template, cell membrane, and the final CMC mimic). The difference in percentage weight loss between the template and the CMC mimic determines the amount of cell membrane-coated onto a template. It also helps to study the stability of the membrane onto a template. The temperature range in TGA depends on the thermal degradation of the samples. For example, the amount and stability of leukocyte membrane coating onto APTES-silica microparticles were demonstrated in the range from 30 to 150 °C. About 15 wt % membrane and 8.0 wt % membrane were observed after 1 and 24 h incubation of CMC mimics in PBS, respectively.^63^ In case of U-251 MG glioblastoma cell membrane-coated magnetic nanocubes (a hybrid of Fe_3_O_4_ and MnO_2_), particles were heated from 100 to 600 °C and observed 12% weight of cell membrane absorbed on nanocubes.^273^ Similarly, for LNCaP-AI prostate cancer cell membrane-coated CaCO_3_ capped mesoporous silica nanoparticles (MSN@CaCO_3_), around 30% weight of cell membrane was absorbed onto MSN@CaCO_3_ after heating samples from 25 to 800 °C.^225^

FT-IR spectroscopy is another versatile technique to qualitatively characterize cell membrane coating by comparing spectra before and after CMC assembly. Only a few reports mentioned this technique to confirm CMC mimics assembly are discussed in this section. The sharp IR characteristics peaks of the template weaken after the cell membrane coating confirms the immobilization of the membranes on the template surface. Along with this, additional strong stretching vibration peaks of C–H/C–N/C=O/N–H/O–H of cell membrane phospholipids, proteins, and carbohydrates are observed if there are no overlaps with the template characteristic peaks itself.

For example, the U-251 MG glioblastoma membrane coating was confirmed onto magnetic nanocubes by observing an additional peak in the range of 1500–3000 cm^–1^.^273^ The additional broad peak at approximately 1750 cm^–1^ was due to the C–N and C=O vibrations of the cell membranes. In the case of MDA-MB-231 breast cancer cell membrane-coated mesoporous silica nanoparticles (SiFePNs), an additional peak was also observed around 2950 cm^–1^ by the C–H band stretching vibration of the methyl group of cell membrane phospholipids and weakening of the sharp IR peaks of SiFePNs after coating.^247^ The characteristic peak of C=O and −NH_2_ in the spectra was reported to confirm the RBC membrane coating onto persistent luminescence nanocarriers.^68^ On occasion, additional peaks can also be attributed due to chemical interaction between the cell membrane and the template surface. For example, in the leukocyte membrane-coated APTES-silica microparticles, two strong peaks were observed at 1652 and 1544 cm^–1^ that correspond to the amide I and II modes of the proteins of the membrane.^63^ The C=O stretching vibrations of the peptide bonds lead to the amide I band, while the C–N stretch coupled with N–H bending mode leads to the amide II band. This amide linkage arose from the peptide bonds between protein residues and the covalent bond between the carboxylic moieties of protein and the primary amines of the APTES molecule in the microparticles. The spectra of CMC mimics also exhibited a weak peak for the Si–O moieties and a strong peak for the C–H stretching compared to that of the APTES-silica particles spectra. This leads to the immobilization of the membranes on the particle’s surface, shielding the silicon surface while exposing the long C–H and C–C chains of phospholipids and proteins.

FRET is another assay used to characterize CMC mimics as they assemble using one or two different cell membranes. In this study, each cell membrane labels with a donor or acceptor fluorescent dye from FRET pairs. 7-Nitrobenz-2-oxa-1,3-diazol-4-yl (NBD) and RhB (rhodamine), DiI, and DiD are the commonly used FRET pairs. This study identifies the molecular distance between the fluorophores using the energy transfer mechanism. Donor energy minimizes when energy transfer occurs from the donor to the acceptor when they are nearby.

For example, fusion of NK cell membrane and liposome to create CMC mimics was validated using a FRET study.^102^ The two sets of liposomes were tagged with fluorescence donor (PE-NBD) and fluorescent acceptor (PE-RhB) and mixed them with NK cell membrane. As the coating progressed, FRET was masked by the NK cell membrane, resulting in a decrease in the fluorescent intensity of the acceptor and an increase in the fluorescent intensity of the donor. Similarly, FRET study has been used to confirm fusion of various cell membranes, for example, the fusion of platelet and RBC membrane using DOPE-RhB/C6-NBD-doped platelet membrane,^97^ fusion of the B16-F10 cancer cell membrane and RBC membrane using DiD/DiO-doped B16-F10 cancer cell membrane,^100^ fusion of liposomes and RBC membrane using DHPE-RhB/C6-NBD-doped liposomes,^239^ and the fusion of RBC and MCF-7 cancer cell membrane using DOPE-RhB/C6-NBD-doped MCF-7 cancer cell membrane.^198^

FRET also helps to validate the fusion of the cell membrane onto a template. In this case, the cell membrane and template are both doped with a FRET pair. For example, the fusion of RBC membrane onto PLGA was confirmed by doping the RBC membrane with NBD-PE and PLGA with RhB-egg phosphatidylcholine (PC).^71^ As the distance between the NBD-labeled RBC membrane and RhB-labeled polymeric core becomes shorter, NBD-energy donor fluorophore becomes more efficient in donating energy to RhB-energy acceptor fluorophore, therefore increasing the fluorescent intensity of the acceptor and decreasing the fluorescent intensity of the donor.

#### Membrane Permeability

The permeability of the cell membrane in the CMC mimics has been little explored. The cell membrane in the live cells is permeable in nature and controls the in and out of the ions using their ion pumps. It helps to understand the better control of the drug encapsulation and release in CMC mimics. The permeability of RBC-PLGA systems was investigated by using the cell membrane permeable molecular probe.^302^ The permeable molecular probe succinimidyl 6-(N-(7-nitrobenz-2-oxa-1,3-diazoyl-4-yl)- amino)hexanoate (NBD-NHS) was used to label both the sides of the RBC membrane and dithionite (S_2_O_4_^2–^) ion for quenching the NBD fluorescence. It was observed that the RBC-PLGA has a higher permeability to the dithionite ions than to living RBC cells and egg-PC/cholesterol liposomes (Figure 5D).

#### Deformability

Deformability is a vital design parameter that affects the behavior of particles on both the micro and nanoscales.^303,304^ It is mostly dependent on the shape and average particle elasticity. Atomic force microscopy (AFM) and compression testing machine/universal testing machines are some of the techniques used for measuring the mechanical properties of CMC mimics. The multiparticle tracking (MPT) method and microfluidic technique help to validate and visualize the deformability of CMC mimics.

**Figure 6:**
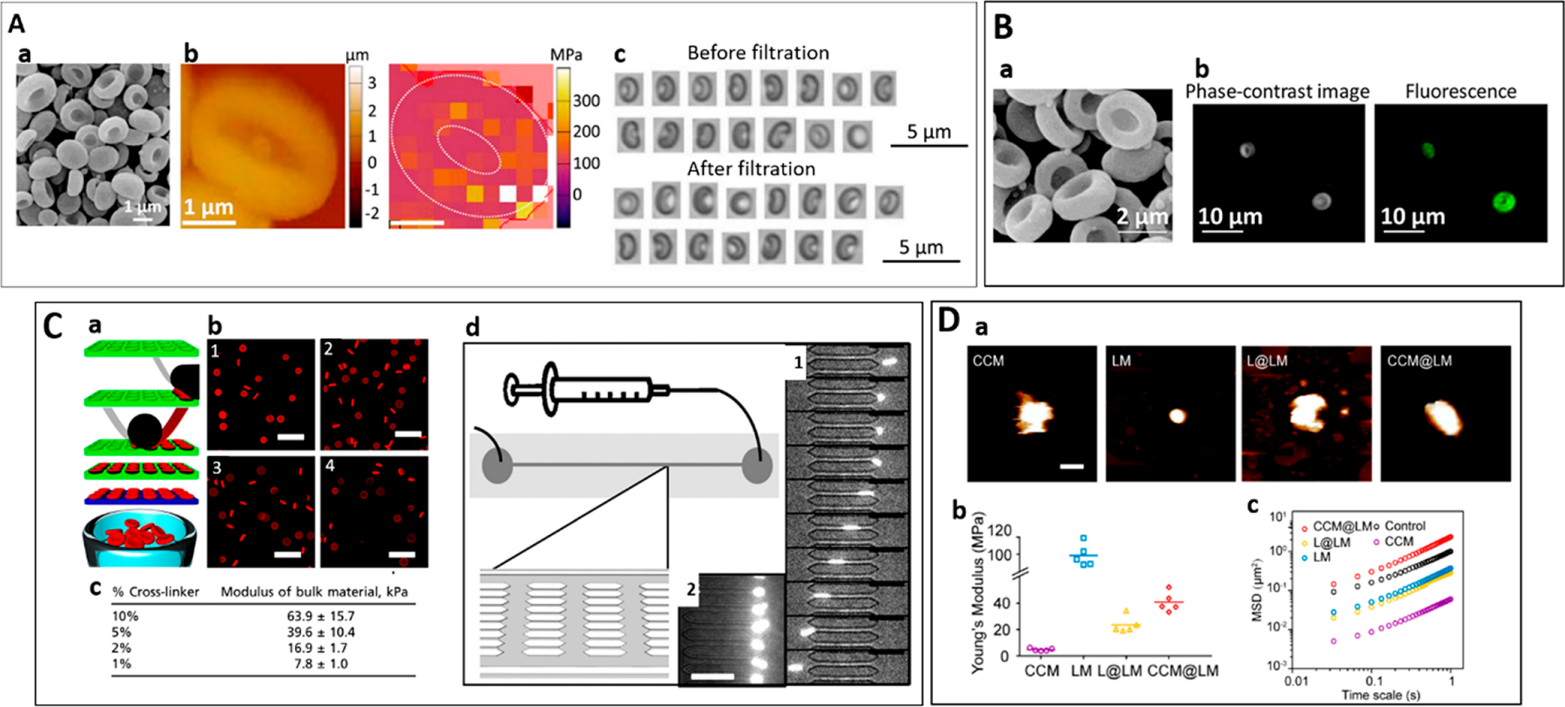
Validation of elastic properties in CMC mimics. (A) RBC-MPs using electrospinning-based technique: (a) SEM, scale bar = 1 μm; (b) IED of RBC-MPs, scale bar = 1 μm; (c) images of RBC-MPs before and after filtration, scale bar = 5 μm. Reprinted with permission from ref (305). Copyright 2018 Elsevier. (B) RBC membrane-coated RBC-MPs mentioned above (a) SEM; (b) phase contrast and CellVue labeled cell membrane-coated RBC-MPs. Reprinted with permission from ref (287). Copyright 2018 American Chemical Society. (C) Disc shaped RBC microparticles (RBCM) replication in: (a) nonwetting templates (PRINT) technique, cross-linked by PEGDA, (b) fluorescence imaging of RBCM varying percent cross-linker: (1) 10% cross-linked, (2) 5% cross-linked, (3) 2% cross-linked, and (4) 1% cross-linked, scale bar = 20 μm; (c) bulk modulus of different cross-linked RBCM; (d) microfluidic evaluation of deformability: (1) 1% cross-linked RBCM and (2) 10% cross-linked RBCM, scale bar = 30 μm. Reprinted with the permission from ref (26). Copyright 2011 United States National Academy of Sciences. (D) MCF-7 breast cancer cell membrane-coated silica nanoparticles-supported PEGylated liposomes: (a) AFM deformation images of CCM, LM (silica nanoparticles-supported PEGylated liposomes), L@LM (1,2-dimyristoyl-*sn*-glycero-3-phosphocholine (DMPC)-coated LM, CM@LM (cancer cell membrane-coated LM), scale bar = 100 nm; (b) Young’s modulus of nanoparticles; (c) MSD values of nanoparticles in a simulated tumor ECM. Reprinted with permission from ref (175). Copyright 2020 American Chemical Society.

There are ongoing efforts to incorporate the elastic properties (Figure 6) in the designed particle system for enhancing mobility and biodistribution in animal studies. For example, RBC-shaped microparticles (RBC-MPs) were prepared using an electrospinning-based technique and showed intraparticle elasticity (IED), which was measured by AFM.^305^ The Young’s modulus (*E*) of the dent in the RBC-MPs was <100 MPa, whereas that of the thick rim was 100–300 MPa. The difference in the *E* value of the dent and the rim in RBC-MPs shaped the particles. This shape helped these particles to deform and retain their original shape after passing through a membrane filter with 1 μm pores. These RBC-MPs also showed less accumulation in the lungs and the spleen. The same group used these RBC-MPs for RBC membrane coating to mimic the shape and the surface structure of RBC for increasing its circulation time in blood.^287^

In 2011, the Prof. DeSimone group also prepared tunable elastic RBC-shaped hydrogel microparticles (RBCMs) in the nonwetting templates (PRINT) technique.^26^ They tested their mechanical properties (bulk modulus) with a Universal Testing Machine (Instron) with a strain rate of 5 mm/min. They achieved tunable modulus of the hydrated polymer from 63.9 to 7.8 KPa by varying the cross-linker from 10% to 1%, respectively, which overlapped with the reported modulus of RBCs (26.7 KPa). They successfully demonstrated the deformability of RBCMs under the flow condition using microfluidic models of vascular constriction. Microfluidics has also widely been used to study the deformability of cells like RBCs,^306^ leukocytes,^307^*etc*. Therefore, microfluidics can also be explored to analyze and visualize the flexibility of CMC mimics.

Recently, the mechanical properties of the yolk–shell structured MCF-7 cancer-cell-membrane-coated mesoporous silica nanoparticles supported liposome (CCM@LM) was validated using AFM and demonstrated using MPT method.^175^ These yolk–shell structures showed moderate rigidity with young’s modulus around 40 MPa. During filtration, these CMC mimics could also transform into an ellipsoidal shape. These properties facilitated its penetration through spheroids *in vitro*. They evaluated its ECM diffusion capability using the MPT method in which the MPT medium was collagen (I) hydrogels. The MSD value of CCM@LM was approximately 7.1- and 2.6-fold higher than that of LM and PLGA nanoparticles, respectively. Incorporating the elastic properties in CMC mimics can enhance its mobility and penetration in the tumors. This property in CMC mimics needs to be explored more in-depth.

### Biological Characterization of CMC Mimics

The cell membrane provides surface functionality to the CMC mimics that allows communication with other cells and helps to escape from macrophages and circulate more in the bloodstream. For example, the CD47 receptor on the RBC membrane selectively binds to signal-regulatory protein alpha (SIRPα) glycoprotein expressed by macrophages to prevent its uptake.^109,110^ Therefore, for CMC mimics to function efficiently, the cell membrane must maintain the right orientation post-coating and contain the maximum amount of translocated protein on its surface. The isolated membrane should have minimal nuclear, mitochondrial, and cytosol contamination, and the transmembrane proteins must face outward for active targeting and accumulation at the intended site. Improper membrane orientation (integral protein exposed onto the other surface of the mimics) will affect cell-to-cell communication, overall function, and risk of macrophage detection and causes unwanted side effects. Therefore, proper qualitative and quantitative evaluation of intact membrane proteins, purity, and orientation is required in CMC mimics to improve their functional efficiency and reproducibility for clinical translation. All the properties are also summarized in Figure 4.

#### Protein Analysis

**Figure 7:**
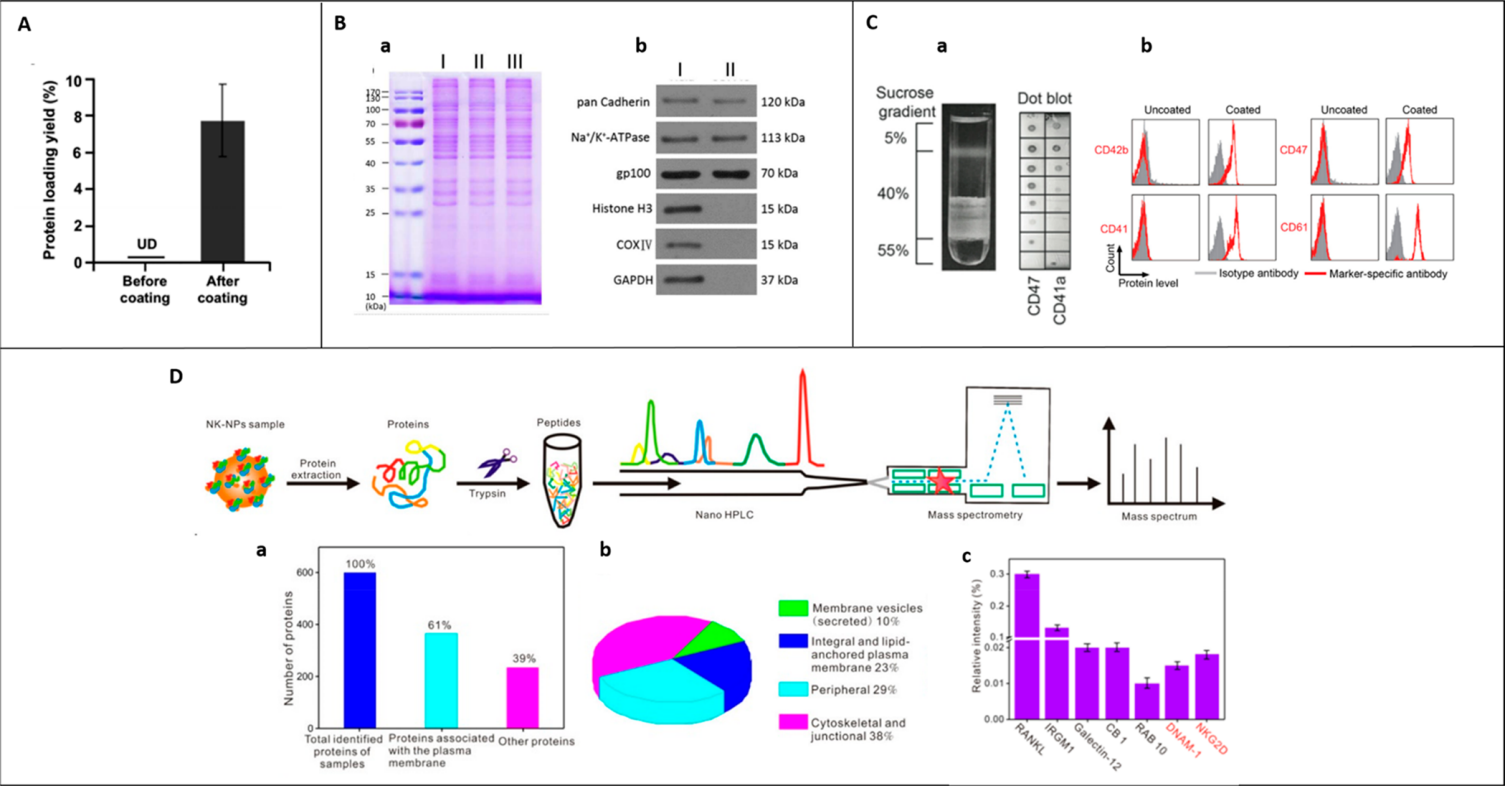
Protein analysis. (A) BCA assay. Quantification of protein concentration of gold nanoparticles before and after bacterial cell membrane coating. Reprinted with permission from ref (94). Copyright 2015 American Chemical Society. (B) Protein profile and identification using (a) SDS PAGE and (b) Western blot on 4T1 breast cancer cell membrane-coated iron oxide nanoparticles. Reprinted with permission from ref (73). Copyright 2016 American Chemical Society. (C) Protein identification using (a) dot blot (CD47, CD41a) in discontinuous sucrose gradient solution and (b) flow cytometry (CD42b, CD47, CD41, and CD61) on platelet membrane-coated APTES-Si (3-aminopropyl triethoxysilane-silica) microparticles. Reprinted with permission from ref (213). Copyright 2016 Elsevier. (D) LC-MS, analysis of human NK cell membrane in NK-NPs (NK cell membrane-coated nanoparticles): (a) the total number of protein and plasma-membrane associated protein detected on NK-NPs, (b) a pie chart of the proteins identified on NK-NPs according to UniProt and GO information and the literature, and (c) flow cytometry analysis to validate the presence of specific proteins on NK-NPs. Reprinted with permission from ref (150). Copyright 2018 American Chemical Society.

The protein composition and expression on the isolated cell membrane and CMC mimics can be analyzed, identified, and quantified using several techniques. These include bicinchoninic acid assay (BCA)/Bradford assay, sodium dodecyl sulfate-polyacrylamide gel electrophoresis (SDS-PAGE), Coomassie brilliant blue staining, Western blot/dot blot, flow cytometry, and liquid chromatography-mass spectroscopy (LC-MS) (Figure 7).

Before the protein analysis, the proteins need to be extracted from the isolated cell membrane and CMC mimics using lysis buffer (for example, radioimmune precipitation (RIPA) buffer). The lysis buffer should be supplemented with protease and phosphatase inhibitor cocktail and phenylmethylsulfonyl fluoride (PMSF) to prevent degradation of proteins and stored at −80 °C for protein analysis. BCA assay and Bradford assay are most commonly used for colorimetric detection and quantification of total protein concentration from various cell membrane and CMC mimics.^65,67,100,150,195,308,309^ Although most of the studies have discussed the use of these assays, there are only a few reports that have mentioned the amount of total protein translocated onto the isolated cell membrane or CMC mimics. In addition, there are no standards reported on total protein content that should be present on CMC mimics for their therapeutic effects. For example, around 300 mg of protein content was found in NK cell (NK-92) membranes extracted from 1 × 10^7^ cells using the Bradford assay.^102^ A 7.9 ± 2.0 wt % protein loading yield of the bacterial cell membrane onto the gold nanoparticles,^94^ 2.8 ± 0.5 wt % protein loading yield of the MIN6 cell membrane onto fibers,^96^ and 18.6 ± 5.7 wt % protein loading yield of the RBC membrane coating onto mesoporous silica nanoparticles^61^ were found using BCA assay. In fact, determination of the protein content before and after the cell membrane coating can also provide an insight into validate the membrane coating onto a template.

The protein profile in the cells, isolated cell membrane, and CMC mimics can be visualized, analyzed, and compared qualitatively by loading and running the same amount of protein in a specific % of SDS-PAGE gel that allows the separation of protein-based on mass.^99,150,207,277^ After the separation of proteins, these gels can be stained with irreversible Coomassie brilliant blue dye. This binds nonspecifically to proteins because of ionic interactions between sulfonic acid groups and positive protein amine groups through van der Waals attractions and appears as blue protein bands.^63,87,106,175,235,310^ The intensity of the blue protein bands helps to compare the total protein profile translocated from a natural cell to the isolated cell membrane and CMC mimics. This is the basic analysis that is reported in almost every literature to validate the successful coating of cell membrane onto a template.

Western blot is the most widely used technique to identify and compare the expression of specific proteins from among a mixture of proteins on the cell lysate, isolated cell membrane, and CMC mimics. For example, using Western blot, comparable presence of CD47 receptor on natural RBC lysate, RBC membrane and RBC membrane-coated Fe_3_O_4_ nanoparticles was observed.^211^ Similarly, comparable DNAM-1, NKG2D, and CD56 (neural cell adhesion molecule receptors) on the NK cell lysate, NK cell membrane, and its membrane-coated PLGA nanoparticle^67^ and mPEG-PLGA nanoparticle^150^ were found, respectively. In the case of hybrid RBC and MCF-7 cancer cell membrane-coated melanin nanoparticles,^198^ comparable RBC-specific membrane proteins (band 3, GPA, CD55, and CD47) and MCF-7 specific membrane proteins (EpCAM, N-cadherin, galectin-3) on hybrid RBC-MCF-7 vesicles and its membrane-coated mimics were also observed. The enrichment of protein-like clusters of differentiation receptors (CD11c, CD86, CD40) expression was found on the dendritic cell membrane and its membrane-coated PLGA nanoparticles than dendritic cell lysate.^106^ Similarly, the significant protein enrichment for LPS binding proteins (CD14, TLR4), cytokine binding receptors (CD126 and CD130 for IL-6, CD120a, and CD120b for TNF, and CD119 for IFN-γ) was observed on macrophage membrane and membrane-coated PLGA nanoparticles than macrophage cell lysate.^130^ Similarly, the enrichment of surface protein like TNF alpha R, IL-1R, LFA-1 receptors was also observed on the neutrophil membrane and its membrane-coated PLGA as compared to the neutrophil lysate.^66^

Dot blot is another blotting technique that requires only a few microliter samples directly onto the PVDF or nitrocellulose membrane followed by blotting procedures. Dot blot is a quick and fast technique used to identify the position of isolated membrane fraction in the sucrose gradient using specific protein markers. For example, leukocyte cell membrane was isolated using 55–40–30% sucrose (w/v) gradient.^63^ The gradient was divided into 10 different fractions, analyzed for the specific markers using dot blot, and found the lipid ring between 40 and 30% sucrose interface (fractions 5 and 6) enriched with plasma membranes. Similarly, a 44–40–5% sucrose gradient was performed and divided the gradient into eight fractions for the dot blot analysis^213^ and found that the majority of platelet membranes were present in the lipid ring between 5 and 40% sucrose interfaces. There are very few reports that have used dot blot to identify the expression of a specific protein on the isolated cell membrane and CMC mimics because it does not provide information on the actual size of the target protein like a Western blot. For example, the presence of CD47 and glycophorin A (GPA) on the RBC membrane and its coated PLGA-Gd nanoparticles was reported using both dot blot and Western blot.^71^ Western blot or dot blot also helps to determine the purity of isolated cell membrane by using nuclear or mitochondrial or cytosol specific antibodies that help to assist in detecting its content in the membranes. For example, histone H3^209,257^ or nucleoporin p62^63^ antibodies as a nuclear marker, cytochrome *c*-oxidase (COX IV)^63,209,257^ or ATP5a^224^ antibody as a mitochondrial marker, and glyceraldehyde-3-phosphate dehydrogenase (GAPDH)^209,224,257^ as cytosol marker and P-cadherin^92^ or Na+/K+-ATPase^73,92,150^ as plasma membrane markers have been used.

Flow cytometry is a powerful qualitative and quantitative technique, useful to identify and quantify specific protein on the CMC mimics by measuring the fluorescent intensity. Only a few reports explore this technique for some protein analysis. For example, the fluorescence intensity of CD42b, CD47, CD41, and CD61 on platelet membrane-coated APTES modified silica nanoparticles^213^ and the fluorescence intensity of CXCR4 on U87 cancer cell membrane-coated PLGA nanoparticles^224^ was identified. An equal amount of LFA-1 receptor on a neutrophil membrane-coated PLGA nanoparticles and neutrophil cells^66^ was observed. Similarly, the comparable fluorescence intensity of MHC-II protein on dendritic cell membrane-coated mimics and natural dendritic cells with an equal amount of MHC-II surface protein was observed.^106^ Therefore, this technique has a lot of potential to analyze and compare the amount of specific protein on CMC mimics with the natural cell in a given batch that may overcome the reproducibility issues.

LC-MS/proteomics analysis is a quantitative large-scale protein study. This study involves fractionation of complex peptide or protein mixtures, acquiring the data necessary to identify individual proteins using mass spectroscopy, and finally, analyzing and organizing the mass spectroscopy data using bioinformatics.^311^ The total number of proteins identified can be further characterized based on their cellular function (integral or peripheral plasma proteins, cytoskeletal or junctional protein), biological process (transport, immunity, cell–cell adhesion, developmental process, proteolysis, lipid metabolism, *etc*.), and molecular function (GTP (guanosine triphosphate) binding, protein binding, GTPase activity, GDP (guanosine diphosphate) binding, actin-binding, *etc*.).^150,239,312^ For example, the shotgun proteomics method identified 868 distinct proteins on human NK cell membrane-coated mPEG-PLGA nanoparticles.^150^ They also analyzed specific proteins on CMC mimics like immunity-related GTPase family M protein 1 (IRGM1), cannabinoid type 1 receptor (CB1), ras-related protein encoded with RAB10 gene (RAB 10), and receptor activator of nuclear factor κ-Β ligand (RANKL) involved in the polarization of M1-macrophages and NKG2D, DNAM-1 in targeting tumor cells. 2215 common proteins were identified on U-251 MG cell membranes and their coated magnetic nanocubes.^273^ Those identified proteins were clusters of differentiation 59 (CD59), epidermal growth factor receptor (EGFR), CD44, tight junction protein 1 (TJP1), myosin light-chain kinase (MYLK), and others. A label-free quantification proteomics method identified 474 membrane proteins on RBC membrane-coated PLGA nanoparticles^313^ and 148 common proteins in erythroliposomes (a hybrid of RBC vesicles and liposomes) and RBC vesicles.^313^ All the research papers reported above have provided a long list of proteins identified on the cell membrane and their CMC mimics.

#### The Orientation of Cell Membranes (Right-Side-Out)

**Figure 8:**
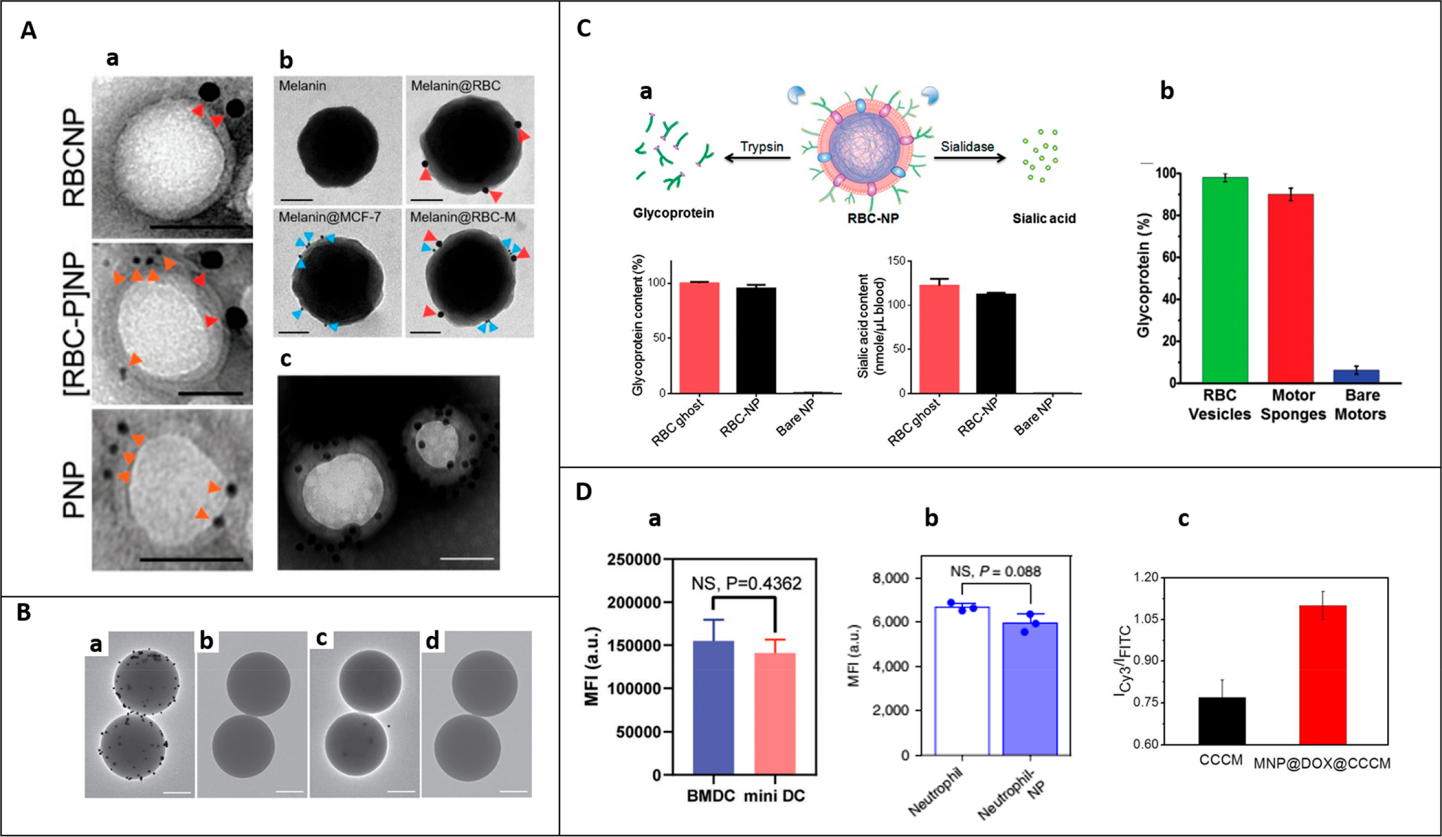
Protein orientation. (A) Immunogold staining: (a) TEM images to identify CD235a (red arrows, large gold) and CD61 (orange arrows, small gold) on RBCNP (RBC membrane-coated PLGA), [RBC-P] NP (hybrid RBC and platelet membrane coated PLGA), and PNP (platelet membrane-coated PLGA). Scale bar = 50 nm. Reprinted with permission from ref (97). Copyright 2017 John Wiley and Sons. (b) TEM images to identify CD47 (red arrow, large gold nanoparticle) and CD340 (blue arrow, small gold nanoparticle) on melanin nanoparticles, melanin@RBC, melanin@MCF-7, melanin@RBC-MCF-7 (hybrid). Scale bar = 50 nm. Reprinted with permission from ref (198). Copyright 2019 Elsevier. (c) TEM image of Si/PNPs@Hela (siRNA/PTX coloaded PLGA nanoparticle) stained with extracellular-domain specific AuNPs-AS1411. Scale bar = 100 nm Reprinted with permission under a Creative Commons CC-BY License from the ref (235). Copyright 2020 Ivyspring International Publisher. (B) Antibody binding assay. TEM images of RBC-AuNPs (RBC membrane-coated gold nanoparticles) with (a) anti-CD47 (exoplasmic) antibody-modified polystyrene microspheres, (b) anti-CD47 (cytoplasmic) antibody-modified polystyrene microspheres, control groups, and AuNPs alone, (c) anti-CD47 (exoplasmic) antibody-modified polystyrene microspheres, and (d) CD47 (cytoplasmic) antibody-modified polystyrene microspheres. Scale bar = 1 μm. Reprinted with permission from ref (286). Copyright 2013 John Wiley and Sons. (C) Sialyl acid and glycoprotein content on the membrane. (a) Comparison of relative glycoprotein content and sialyl acid recovered from RBC ghost, RBC-NPs (RBC-coated PLGA nanoparticles), bare NPs (PLGA). Reprinted with permission under a Creative Commons CC-BY License from ref (300). Copyright 2014 Royal Society of Chemistry. (b) Glycoprotein content on RBC vesicles, motor sponges (RBC membrane-coated gold nanowires), and bare motors (gold nanowires). Reprinted with permission from ref (299). Copyright 2015 John Wiley and Sons. (D) Transmembrane and internal membrane proteins identification. (a) Transmembrane fluorescence intensity (PE-labeled antimouse MHC II antibody) of bone marrow-derived dendritic cells (BMDC) and mini DC (mature dendritic cell membrane-coated PLGA). Reprinted with permission under a Creative Commons CC-BY License from ref (106). Copyright 2020 Wiley-VCH. (b) Transmembrane fluorescence intensity comparison measured in neutrophils and neutrophils-NPs (membrane-coated PLGA nanoparticles) stained with allophycocyanin antimouse LFA antibodies. Reprinted with permission from ref (66). Copyright 2018 Springer Nature. (c) Fluorescence intensity ratios after immunofluorescence staining of intracellular (IgG-FITC) and extracellular domains (IgG-Cy3) of CXCR4 receptor on 4T1 cancer cell membrane (CCCM) and cell membrane-coated on magnetic iron oxide nanoparticles loaded with doxorubicin (MNP@DOX@CCCM). Reprinted with permission from ref (73). Copyright 2016 American Chemical Society.

The orientation of the cell membrane in the CMC mimics helps in determining the direction of the receptors while coating onto a template. The extracellular proteins must be directing outward, whereas the intracellular proteins should be directing inward, to maintain the functionality of the CMC mimics. Among the techniques/method for studying the orientation are immunogold staining, antibody binding assays, quantification of glycoproteins or sialic acid content, and quantification of transmembrane or internal membrane proteins (Figure 8).

Immunogold staining or labeling is a qualitative technique used in electron microscopy for the identification and distribution of a specific protein of interest located on the interior or the exterior of the cell membrane of the CMC mimics. It utilizes gold nanoparticles conjugated with the secondary antibody of interest and, in turn, attaches to the primary antibody designed to bind a specific protein on the CMC mimic. The gold nanoparticles are visible as a black spot under electron microscopy and help visualize the arrangement of a specific protein onto CMC mimics. Some of the reported specific biomarkers on RBC membrane are CD47^198^ and CD235a,^97^ platelet membranes are CD61^97^ and CD47,^93^ and MCF-7 breast cancer cell membrane is CD340^198^ that have been used in the immunogold staining and analyzed under TEM. The orientation of the HeLa membrane onto PLGA nanoparticles was also demonstrated using immunogold staining, but tagged the gold nanoparticles with AS1411, a nucleic acid aptamer that targets the extracellular region of nucleolin in the cell membrane and is analyzed under a transmission electron microscope.^235^

Antibody binding assay was reported to examine the coating and sidedness of RBC membrane onto gold nanoparticles.^286^ Two distinct anti-CD47 antibodies (exoplasmic and cytoplasmic) conjugated polystyrene microspheres were used for the interaction with the membrane and further analyzed under TEM.

Glycans and sialic acid groups are asymmetrically distributed on the extracellular side of the cell membrane. Hence, their quantification has also been reported for validating the sidedness of the RBC membrane in a CMC mimic.^211,299,300^ The glycoprotein content and terminal sialic groups were first enzymatically removed from CMC mimics using trypsin and sialidase, respectively. Further, the glycoprotein content was quantified using a periodate-based glycoprotein detection assay or mouse glycoprotein ELISA kit and sialic acid content using a sialic acid quantification kit.

Transmembrane protein and/or internal membrane protein in a CMC mimic can also be identified, quantified, and compared with the cell source using flow cytometry or immunoblotting or fluorescence microscopy to validate the orientation of the cell membrane. The localization of CD3z (intracellular) and LFA-1 (extracellular) proteins on leukocyte membrane-coated mimics was determined using both flow cytometry and immunoblotting. The fluorescence intensity of LFA-1 on CMC mimics was observed to be three times higher than that of the leukocyte vesicles but detected CD3z signals only after permeabilization.^63^ The comparable intensity of major histocompatibility complex II (MHC-II) protein (extracellular) on dendritic cell membrane-coated mimics and dendritic cells was confirmed using flow cytometry.^106^ Similarly, the comparable intensity of LFA-1 (extracellular) protein on neutrophils membrane coated mimics and neutrophils cell^66^ and comparable intensity of CD47 antibody (extracellular) on RBC membrane-coated mimics and RBC ghosts was observed using flow cytometry.^278^ In the case of platelet membrane-coated mimics, two anti-CD41 (glycoprotein (Gp) IIb/IIIa integrin) primary antibodies were used for binding to the N-terminal and C-terminal of CD41 present on the extracellular and intracellular regions, respectively.^213^ Extracellular CD41 intensity was observed four times higher than that in its intracellular domain on the outer surface of CMC mimics using flow cytometry. Similarly, for UM-SCC-7 membrane (human squamous carcinoma cell line) and its mimics, two anti-CXCR4 antibodies were used to bind the extracellular and intracellular region.^73^ A comparable fluorescence intensity of CXCR4 on CMC mimics and cells using a fluorometer was observed. Epifluorescence microscopy has also been reported to analyze the immunostained CD3 receptor (extracellular) on T cell membrane-coated mimics.^280^ Fluorescence intensity per particle using ImageJ software was quantified and observed that 40% of the CMC mimics displayed some or all the CD3 receptors in the correct orientation.

### Role of CMC Mimics in Various Therapeutic Applications

**Figure 9:**
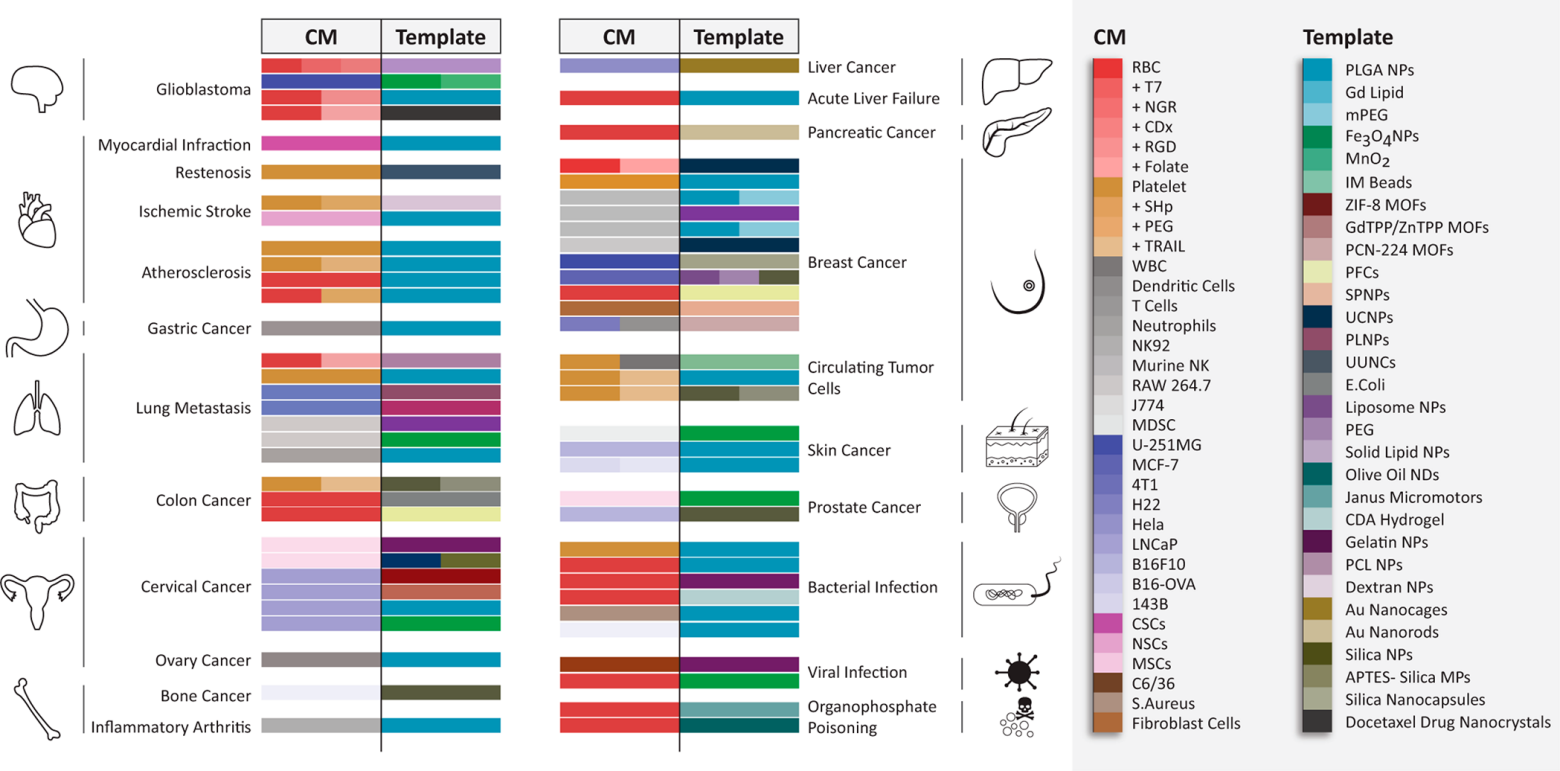
Schematic overview of the types of CMC mimics used for a wide variety of applications. Designs of CMC mimics have utilized combinations of a template and cell membrane that endows the final system with specific therapeutic properties. We have grouped and color-coded them based on their type and modifications. Abbreviations/cell line: CM, cell membrane; RBC, red blood cell; WBCs, white blood cells; NK, natural killer; CSCs, cardiac stem cells; NSCs, neural stem cells; MDSCs, myeloid-derived suppressor cells; TRAIL, TNF-related apoptosis-inducing ligand; RGD, arginylglycylaspartic acid; SHp, stroke homing peptide; APTES, 3-aminopropyl triethoxysilane; PLGA, poly(lactic-*co*-glycolic acid); Fe_3_O_4_, iron oxide; MnO_2_, manganese oxide; Au, gold; GdTPP, gadolinium porphyrin; ZnTPP, zinc porphyrin; PCN-224, porphyrin (TCPP)-based Zr_6_ cluster; PFCs, perfluorocarbons; UCNPs, upconversion nanoparticles; PLNPs, persistent luminescent nanoparticles; MOFs: metal–organic frameworks; ZIF-8, zeolitic imidazolate framework; UUNCs, ultrasmall unimolecular nanocluster; NPs: nanoparticles; NDs, nanodroplets; SPNPS: semiconducting polymer nanoparticles; IM, immunomagnetic; PCL, polycaprolactone; CDA, cystine dimethacrylate; PEG, poly(ethylene glycol); mPEG, methoxy polyethylene glycol; NK-92, human natural killer cell line; RAW 264.7, murine macrophage cell line; J774, murine macrophage cell line; U-251 MG, glioblastoma multiforme cell line; MCF-7, human breast cancer cell line; 4T1, human breast cancer cell line; H22, murine hepatocarcinoma cell line; HeLa, human cervical cancer cell line; B16-F10, murine melanoma cell line; B16.OVA, B16F10 cells expressed ovalbumin; 143B, human osteosarcoma cell line; LNCaP-AI, prostate cancer cell line; *S. aureus*, *Staphylococcus aureus*; *E. coli:*, *Escherichia coli*; C6/36, mosquito medium host cell line (*Aedes albopictus*).

CMC mimics have gained attention in several therapeutic applications like cancer, inflammatory diseases, and infectious diseases. The purpose of designing these mimics is to achieve target efficacy and accumulation at the target site. Figure 9 presents an overview of the different cell membrane and template combinations used for applications, along with a summary of *in vitro* and *in vivo* models used to validate their efficacy in Table 3.

**Table 3:** Summary of *In Vitro* and *In Vivo* Models Used for Validating the Efficacy of CMC Mimics with/without Modification in Various Treatments^a^

cell membrane source/modification	template	encapsulation	*in vitro* models	*in vivo* models	mode of administration	treatment	ref
platelets	PLGA nanoparticles	vancomycin	MRSA252, THP-1, HUVECs	male CD-1 mice (25g), MRSA252	intravenous	MRSA bacterial infection	(93)
	PLGA nanoparticles	docetaxel	–	male Sprague–Dawley rats (300–350 g), angioplasty-induced arterial denudation	intravenous	restenosis	(93)
	PLGA nanoparticles	–	antiplatelets antibodies	male CD-1 mice (6 week old, 20–24 g)	intraperitoneal	immune thrombocytopenia purpura	(212)
	PAMAM-PVL ultrasmall unimolecular nanoparticles	endothelium-protective epigenetic inhibitor (JQ1), or Rapamycin	–	Male Sprague–Dawley rats (∼400 g), carotid artery balloon angioplasty	intravenous	restenosis	(122)
	PLGA nanoparticles	–	J774, HUVECs	ApoE knockout mice	intravenous	Atherosclerosis	(123)
	PLGA nanoparticles	doxorubicin, Indocyanine green	MCF-7, MDA-MB-231, CAMA-1, HeLa	female nude mice, MDA-MB-231 (footpad)	intratumoral	circulating tumor cells of breast cancer Lung metastasis of breast cancer	(87)
				female nude mice, MDA-MB-231 (back)	intravenous		
				female nude mice, MDA-MB-231-luc (orthotropic)	intravenous		
	PLGA nanoparticles	verteporfin	4T1, NIH-3T3	BALB/c mice (3–5 week old), 4T1	intravenous	breast cancer	(121)
platelets/RGD	melanin nanoparticles	doxorubicin	RAW264.7, HUVECs, MDA-MB-231, MDA-MB-231/ADR	Female BALB/c nude mice (18–20 g), MDA-MB-231/ADR	intravenous	lung metastasis of breast cancer	(408)
platelets/TRAIL	acrylamide nanogels	doxorubicin	MDA-MB-231, RAW264.7	Sprague–Dawley (SD) rats (200 ± 20 g), MDA-MB-231	intravenous	circulating tumor cells of breast cancer Lung metastasis of breast cancer	(339)
platelets/TRAIL	silica microparticles-APTES	–	MDA-MB-231, PC3, THP-1	C57BL/6 mice (6–8 week old), MDA-MB-231,COLO 205, B16-F10, MC38	intravenous	circulating tumor cells of breast cancer	(213)
			microrenathane microtubes coated human fibrinogen (thrombosis model)				
platelets + WBCs	immunomagnetic beads	–	MCF-7, HCT116, HeLa	–	–	circulating tumor cells of breast cancer	(99)
platelets + RBC	gold nanowire	–	MRSA USA300, α-toxins	–	–	*S. aureus* bacterial infection	(196)
platelets + RBC	PLGA nanoparticles	–	MDA-MB-231, HFF-1 foreskin fibroblasts, THP-1	ApoE knockout mice	intravenous	atherosclerosis	(97)
RBC	PLGA nanoparticles	vancomycin	MRSA USA300, THP-1, α-toxins	–	–	MRSA bacterial infection	(114)
	PLGA nanoparticles	rapamycin	RAW264.7	ApoE knockout mice (8 week old, 25–30 g)	intravenous	atherosclerosis	(211)
	olive oil nanodroplets	–	organophosphates (paraoxon, diisopropyl fluorophosphate, dichlorvos)	ICR mice (6 week old)	intravenous, intraperitoneal	organophosphate poisoning	(77)
	PLGA nanoparticles	–	HUVECs, α-toxins, streptolysin-O, melittin	female nu/nu nude mice (6 week-old), α-toxins	subcutaneous	pore forming toxins	(115)
	PLGA nanoparticles	mesenchymal stem cell, growth factors	THLE-2, HSC-T6, mouse lung cells, human primary M1 macrophages	C57BL/6 mice (18–25 g)	intravenous	acute liver failure	(86)
	Human serum albumin nanoparticles	Perfluorotributylamine, indocyanine green	HeLa, CT-26, RAW264.7	male BALB/C mice (5–6 week), CT26	intravenous	colon cancer	(269)
	Fe_3_O_4_ nanoclusters	–	MCF-7, RAW264.7	female nude mice (4 week old), MCF-7	intravenous	Breast cancer	(265)
	*E. coli*	–	primary macrophages	female BALB/C mice (6–8 week old), CT26 and 4T1	intravenous	colon cancer, breast cancer	(59)
	PLNPs (Zn_1.25_Ga_1.5_Ge_0.25_O_4_:Cr^3+^,Yb^3+^,Er^3+^) coated with SiO_2_	–	Balb/3T3, HeLa, MCF-7, 4T1, RAW264.7	BALB/c mice, 4T1	intravenous	breast cancer	(68)
	gelatin nanoparticles	vancomycin	RAW 264.7, *S. aureus*, *E. coli*, *S. epidermidis*, *P. vulgaris*, *S. marcescens*, *P. aeruginosa*	–	–	Gram positive bacterial infection	(64)
	gold nanorods	–	Capan-2	male Balb/c nude mice (6–8 week old), Capan-2	intravenous	pancreatic ductal adenocarcinomas	(261)
	PLGA nanoparticles	–	HUVEC, mouse RBCs	Male CD-1 mice (8 week old)	intravenous	MRSA bacterial infection	(278)
	PLGA nanoparticles	–	anti-RBC polyclonal IgG	CD-1 mice, anti-RBCs	intravenous	autoimmune anemia	(409)
	PLGA nanoparticles	Fe_3_O_4_ nanoparticles	influenza virus, Mardin Darby canine kidney cells	–	–	influenza virus	(74)
	carbon nanotube-based field effect transistor	–	MRSA USA300, Pore forming toxins (melittin, streptolysin O, Hlα)	–	–	MRSA bacterial infection	(75)
RBC/cRGD peptide	docetaxel nanocrystal	–	HUVECs, U87	male nude mice (20–22 g), U87, antisubcutaneous tumor- and antiorthotropic glioma	intravenous	glioblastoma	(333)
			BBTB model (HUVEC/U87 coculture)				
RBC/T7, NGR peptide	solid lipid nanoparticles	–	HUVECs, bEnd.3, C6	Tg (fli1:egfp) strain of zebrafish, ICR mice (22–24 g), C6	intracranial	glioblastoma	(335)
			BBB model(upper: bEnd.3)				
			BBTB model (upper: HUVECs, lower: C6)				
RBC/anti-EGFR-iRGD	–	paclitaxel	MKN45	male BALB/c nude mice weighing 18–20 g (5–6 week old), MKN45	intravenous	gastric cancer	(332)
RBC/iRGD	polycaprolactone nanoparticles	paclitaxel	4T1, RAW264.7	Balb/c mice (18–20 g), orthotopic 4T1	Intravenous	Lung metastasis of breast cancer	(309)
RBC/folate receptor	UCNPs (β-NaYF_4_:Er^3+^,Yb^3+^)	–	MCF-7	BALB/c nude mice, MCF-7	intravenous	breast cancer	(277)
RBC/ ^D^CDX peptide	PLGA nanoparticles	doxorubicin	primary brain capillary endothelial cells	nude mice, U87	intravenous	glioblastoma	(234)
			BBB model (Wistar rat primary brain capillary endothelial cells)				
RBC/stroke homing peptide (SHp)	boronic acid conjugated dextran nanoparticles	NR2B9C	PC-12, BBB model (rat brain capillary endothelial cell line)	male Sprague–Dawley (SD) rats (5–6 week, 200 ± 20 g)	intravenous	ischemic stroke	(410)
RBC+ MCF-7	Melanin nanoparticles	-	MCF-7, 4T1, MCF-10A, RAW264.7	female athymic Balb/c nude mice (20–22 g), MCF-7	intravenous	breast cancer	(198)
NK-92	poly(ethylene glycol) methyl ether-*block*-PLGA nanoparticles	4,4′,4″,4‴-(porphine-5,10,15,20-tetrayl) tetrakis (benzoic acid) (TCPP)	4T1, MCF-7, MCF10A	female BALB/c mice (6 week old, 18–22 g)	intravenous	breast cancer	(150)
	liposomes	doxorubicin	MCF-7, NHost	female nu/nu nude mice (6 week old), MCF-7	intravenous	breast cancer	(102)
neutrophils	PLGA nanoparticles	carfilzomib	HUVECs, 4T1, 4T1 (GFP+ or luc+)	female Balb/c nude mice (20 ± 2 g), GFP+4T1, luc+4T1	intravenous	lung metastasis of breast cancer	(55)
	PLGA nanoparticles	–	HUVECs, primary human chondrocytes	human TNF-α male transgenic mice (5 week old) DBA/1J mice in collagen-induced arthritis (6 week old, 18–22 g)	intra-articular	inflammatory arthritis	(66)
natural macrophage cells	gold nanoshells	–	4T1	male Balb/c nude mice, 4T1	intravenous	breast cancer	(139)
	silica nanocapsules	doxorubicin	RAW 264.7, NIH/3T3, 4T1	male BALB/c nude mice, 4T1	intravenous	breast cancer	(69)
RAW 264.7	liposomes	emtansine	RAW 264.7, 4T1, 4T1-luc	female BALB/c nude mice (18–22 g), 4T1-luc	intravenous	lung metastasis of breast cancer	(129)
	Fe_3_O_4_ nanoparticles	–	RAW 264.7, MCF-7	Female BALB/c nude mice (6–8 week old), MCF-7	intravenous	breast cancer	(131)
	UCNPs (β-NaYF_4_:Er^3+^,Yb^3+^ rare earth)	–	RAW 264.7, MCF-7	female BALB/c nude mice (6–8 week old), MCF-7 male ICR mice (6–8 week old)	intravenous	breast cancer	(132)
	bismuth selenide nanoparticles	quercetin	RAW 264.7, 4T1, HUVECs	BALB/c mice, 4T1	intravenous	lung metastasis of breast cancer	(88)
			transwell cell invasion (upper: 4T1)				
			wound healing (4T1)				
			dual-chamber transwell (5 μm, 0.4-μm sized microporous membranes)				
J774	PLGA nanoparticles	–	J774, HEK-blue mTLR4, HUVECs	male BALB/c mice (6 week old), LPS (endotoxemia model)	intravenous	sepsis	(130)
U937	PLGA nanoparticles	–	MCF-7	–	–	breast cancer	(298)
human cytotoxic T-lymphocyte	PLGA nanoparticles	paclitaxel	MKN-45, differentiated THP-1	male Balb/c nude athymic mice (4–6 week old), MKN-45	intravenous	gastric cancer	(98)
CD4^+^ T cells	PLGA nanoparticles	–	HIV stains (X4 and R5)	–	–	HIV	(159)
dendritic Cells	PLGA nanoparticles	IL-2	primary CD8^+^ T, T cells, NIH-3T3, 293T, ID8	C57BL/6 mice (6–8 week old), ID8	subcutaneous	ovarian cancer	(106)
bone marrow-derived dendritic cells +4T1	PCN-224 MOFs	–	4T1, CT26, 3T3	Balb/c mice, 4T1	intravenous	breast cancer	(168)
Human adiposed- derived stem cells	PLGA nanoparticles	–	J774, THP-1	female C57BL/6 mice (10 week old, 22–24 g)	intravenous	hindlimb ischemia	(227)
			transwell: HUVECs				
adipose-derived mesenchymal stem cells	Fe_3_O_4_ nanoparticles	–	TRAMP-C1, RAW246.7	–	–	prostate cancer	(95)
cardiac stem cell	PLGA microparticles	cardiac stem cells growth factors	cardiomyocytes	male SCID Beige mice	intramyocardial	myocardial infarction	(187)
bone marrow derived mesenchymal stem cell membrane	gelatin nanogels	doxorubicin	HeLa, L02	female BALB/c nude mice (∼4 week old), HeLa	intravenous	cervical cancer	(186)
	UCPNs (β-NaYF_4_:Er^3+^,Yb^3+^ rare earth)	–	HeLa, L02	female BALB/c nude mice (∼4 week old), HeLa	intravenous	cervical cancer	(185)
neural stem cell/CXCR4	PLGA nanoparticles	–	–	male C57BL/6 mice (≈20 g)	intravenous	ischemic stroke	(188)
B16-OVA/mannose	PLGA nanoparticles	imiquimod, R837 (toll-like receptor 7 (TLR-7) agonist	bone marrow-derived dendritic cells	female BALB/c mice (6–8 week old), B16-OVA	Intradermal	melanoma	(343)
U87/CXCR4	PLGA nanoparticles	–	U87, U87-CXCR4, MDA-MB-231, BT-474, HMFs	female athymic Balb/c (nu/nu) mice, Female immunocompetent Balb/c mice(6–8 week old)	intravenous	lung metastasis of breast cancer	(224)
			transwell model: HMFs				
LNCaP-AI	CaCO_3_ capped mesoporous silica nanoparticles	doxorubicin	LNCaP-AI, QSG-7701, MCF-7	BALB/c nude mice (6 week old), LNCaP-AI	intravenous	prostate cancer	(225)
HeLa	Fe_3_O_4_ nanoparticles	doxorubicin	HeLa, UM-SCC-7, COS7, RAW 264.7	female BALB/c nude mice (4–5 week old), UM-SCC-7	intravenous	cervical cancer	(173)
	PLGA nanoparticles	paclitaxel, small interfering RNA (siRNA-E7)	HeLa, Ect1, LO2, RAW264.7	female BALB/c nude mice (4–5 week old, 18–22 g), HeLa	intravenous	cervical cancer	(235)
	GdTPP and ZnTPP nanocomposites	–	HeLa	nude mice, HeLa	intravenous	cervical cancer	(219)
	ZIF-8 MOFs	catalase, Al(III) phthalocyanine chloride tetrasulfonic acid	4T1, HeLa, SCC-7, COS7, RAW264.7	BALB/c-nu mice (4–5 week old), HeLa	intravenous	cervical cancer	(342)
143B	silica nanoparticles	indocyanine green	143B	female Balb/c nude mice (6–8 week old), 143B	intravenous	bone cancer	(174)
MCF-7	PLGA nanoparticles	indocyanine green	MCF-7, 293T, MCF-10A, HepG2, A549, MDA-MB-231	female BALB/c mice (4–6 week old), MCF-7	intravenous	breast cancer	(65)
	mesoporous silica nanoparticles supported with PEGylated liposomes	–	SK-hep-1, Caco-2, HeLa, Bxpc-3, Huh-7, MCF-7	BALB/c nude mice (20g), MCF-7	intravenous	breast cancer	(175)
			3D- spheroids of MCF-7				
MDA-MB-435, B16-F10	PLGA nanoparticles	–	bone marrow-derived DCs, MDA-MB-435, B16-F10, HFFs	female C56BL/6 mice (6 week old), MDA-MB-435 Female C56BL/6 mice (6 week old), B16-F10	intradermal	melanoma	(92)
4T1	poly(caprolactone) nanoparticles	paclitaxel	RAW264.7, WML2, 4T1, MDA-MB-435, A549, BEL-7402	female BALB/c nude mice, 4T1, 4T1-luc, female BALB/c mice (4–5 week old, 18–22 g), 4T1	intravenous	lung metastasis of breast cancer	(220)
	gold nanocages	doxorubicin	4T1	BALB/c nude mice (18–22 g) orthotopic, 4T1	intravenous	lung metastasis of breast cancer	(221)
	PLNPs	–	4T1	female BALB/c nude mice (4 week old, 18–22 g), 4T1	intravenous	lung metastasis of breast cancer	(72)
	carbon sphere dotted with cerium oxide nanoparticles	–	COS7, 293T, 4T1	female BALB/c mice (6 week old), 4T1	intravenous	breast cancer	(297)
SGC7901	silica nanoparticles	chlorins e6	SGC7901	female BALB/c nude mice (6–8 week old), SGC7901	intravenous	gastric cancer	(174)
U-251 MG	Fe_3_O_4_ nanoparticles	–	U-251 MG, SH-SY5Y, C8-D1A, bEnd.3	–	–	glioblastoma	(273)
			U-251 MG 3D spheroids				
			BBB model (upper chamber: bEnd.3; lower chamber: U-251 MG)				
HNSCC	gelatin nanoparticles	cisplatin	patient-derived tumor cell	BALB/c nude mice (male, 6–8 week old), patient derived tumor cells	intravenous	head and neck squamous cell carcinoma	(105)
K562	Fe_3_O_4_ nanoparticles	doxorubicin, indocyanine green	MG-63, A549, SW380, A375, HepG2, MCF-7, MGC-803, L02, RAW 264.7	BALB/c nude mice, MG-63	intravenous	osteosarcoma	(103)
MDA-MB-435, DU145, CAL27, HCT116	UCNPs (β-NaYF_4_:Er^3+^,Yb^3+^)	–	RAW264.7, MDA-MB-435, DU145, CAL27, HCT116	female BALB/c nude mice (6–8 week old), MDA-MB-435	intravenous	breast cancer	(223)
B16.OVA, B16.F10, LL/2, CMT64.OVA, MB49, A549, SKOV-3	adenovirus serotype 5 particles	–	A549, SKOV-3	female C57BL/6 mice (4–6 week old) B16.OVA, B16.F10, LL/2,	intratumoral	melanoma lung cancer	(214)
H22	gold nanocages	doxorubicin	RAW 264.7, H22	Sprague–Dawley (SD) male rats (6–8 week old) male BALB/c mice (6 week old, 20–22 g), H22	intratumoral; intravenous	liver cancer	(226)
AF	semiconducting polymer (poly(cyclopentadithiophenealt-benzothiadiazole) nanoparticles	–	AF, fibroblasts, chondrocytes, 4T1, HeLa, SKOV3	female NCr nude mice (4–6 week old), 4T1	intravenous	breast cancer-associated fibroblasts	(104)
MIN6	polycaprolactone nanofibers	–	MIN6	–	–	β-cell proliferation and function	(96)
*E. coli* DH5α + B16-F10	polydopamine nanoparticles	–	B16-F10, NHDF, MCF-7, A549	female C57BL/6 mice (6–8 week old), B16-F10	intravenous	melanoma	(200)
*E. coli* K12/ *Rhizobium etli* tyrosinase	–	melanin	4T1	female athymic Fox-N-1 nude mice (6 week old), 4T1; female C57BL/6 mice (6 week old)	intravenous, intratumoral	breast cancer	(194)
*E. coli*	Gold nanoparticles	–	–	Male CD-1 mice (6 week old), *E. coli*	subcutaneous	*E. coli* bacterial infection	(94)
*S. aureus*	PLGA nanoparticles	Vancomycin or Rifampicin	Ana-1, NIH-3T3	Balb/c mice ((5–6 week old), *S. aureus*	intravenous	*S. aureus* bacterial infection	(192)
CRKP	BSA nanoparticles	–	RAW 264.7, DC2.4	female C57BL/6 mice (6–8 week old)	subcutaneous	CRKP bacterial infection	(193)
C6/36	gelatin nanoparticles	–	Vero, HeLa, HEK 293T	type I interferon (IFN-α/β) receptor deficient (A129) mice	intravenous	Zika viral infection	(228)
293T/ACE2 + THP-1	–	–	Vero-E6, Caco-2	ICR mice (20 to 25 g, 4- 6 week old)	intratracheal	SARS-CoV-2 infection	(397)
NL-20, THP-1	PLGA nanoparticles	–	Vero E6	C57BL/6NHsd mice	intratracheal	SARS-CoV-2 infection	(400)
mitochondria (mouse liver)	PLGA nanoparticles, carbon nanotube-based field-effect transistors (FET)	–	ABT-263, HL-60	female C57BL/6 mice (6 week old)	oral gavage	ABT-263-induced thrombocytopenia	(91)

a Abbreviations/cell lines: APTES, 3-aminopropyl triethoxysilane; PAMAM-PVL, poly(amidoamine)-polyvalerolactone; PBMC, peripheral blood mononuclear cells; SHp, stroke homing peptide; BSA, bovine serum albumin; UCNPs, upconversion nanoparticles; PLNPs, persistent luminescent nanoparticles; MOFs, metal–organic frameworks; ZIF-8, zeolitic imidazolate framework; PCN, porous coordination network; MSCs, mesenchymal stem cells; HMFs, human mammary fibroblasts; HFFs, human foreskin fibroblasts; HNSCC, head and neck squamous cell carcinoma; AF, activated fibroblast; DCs, dendritic cells; CXCR4, C–X–C chemokine receptor type 4; MRSA252, methicillin-resistant *S. aureus*; THP-1, human acute monocytic leukemia cell line; HUVECs, human umbilical vein endothelial cell line; RAW 264.7, murine macrophage cell line; J774, murine macrophage cell line; MCF-7, human breast cancer cell line; 4T1, human breast cancer cell line; 4T1-Luc, 4T1 cells expressing luciferase; HeLa, human cervical cancer cell line; MDA-MB-231, breast cancer cell line; MDA-MB-231/ADR, DOX-resistant breast cancer cell line; 3T3, murine normal fibroblast cell line; CT26, murine colon cancer cell line; NIH-3T3, fibroblast cell line; ID8, murine ovarian surface epithelial cell line; MKN-45, human gastric cancer cell line; CAMA-1, human breast cancer cell line; CAL 27, human squamous carcinoma cell line; RD, human rhabdomyoma cell line; MKN45, gastric cell line; Capan-2, human pancreatic cancer cell line; C6, glioma cell line; 143B, human osteosarcoma cell line; SCC-7, murine squamous cell carcinoma cell line; Ect1, endocervical cell line; L02, normal hepatic cell line; LNCaP-AI, prostate cancer cell line; QSG-7701, human normal liver cell line; UM-SCC-7, head and neck squamous cell carcinoma cell line; HCT116, human colorectal cancer cell line; TRAMP-C1, mouse prostate cancer cell line; COS7, african green monkey kidney cell line; 293T, human embryonic kidney cell line; WML2, murine lung fibroblast cell line; BEL-7402, human hepatoma cell line; SK-hep-1, human hepatic adenocarcinoma cell line; Caco-2, human colorectal adenocarcinoma cell line; Bxpc-3, human pancreatic cancer cell line; Huh-7, human liver cell line; MCF-10A, nontumorigenic epithelial cell line; U-251 MG, glioblastoma multiforme cell line; SH-SY5Y, neuroblastoma cell line; C8-D1A, astrocytes type 1 clone; bEnd.3, mouse brain endothelial cell line; MDA-MB-435, human breast carcinoma cell line; DU145, human prostate cancer cell line; HCT116, human colorectal cancer cell line; CAL27, human squamous cancer cell line; A375, human melanoma cell line; HepG2, human hepatocellular carcinoma cell line; K562, human myelogenous leukemia cell line; MGC-803, human gastric carcinoma cell line; MG-63, human osteosarcoma cell line; L02, human hepatic cell line; SW380, human colorectal adenocarcinoma cells; B16-F10, murine melanoma cell line; B16.OVA, B16F10 cells expressed ovalbumin; LL/2, murine lewis lung carcinoma cell line; CMT64.OVA, lung carcinoma cell line expressed ovalbumin; MB49, murine bladder carcinoma cell line; A549, human nonsmall cell lung cancer cell line; SKOV-3, ovarian cancer cell line; CAL27, human squamous cancer cell line; H22, murine hepatocarcinoma cell line; MIN6, pancreatic β-cell line; C6/36, *A. albopictus* (mosquito medium host cell line; *E. coli*, *Escherichia coli*; *E. coli*K12, *Escherichia coli* derived from W3110; CRKP, carbapenem-resistant *Klebsiella pneumonia*; *S. aureus*, *Staphylococcus aureus*; *P. aeruginosa*, *Pseudomonas aeruginosa*; *S. marcescens*, *Serratia marcescens*; *P. vulgaris*, *Proteus vulgaris*; *S. epidermidis*, *Staphylococcus epidermidis*; Ana-1, murine macrophage cell line; DC2.4, murine dendritic cell line; NL-20, human lung epithelial cells; HL-60, human leukemia cell line; ABT-263, B-cell lymphoma 2 (Bcl-2) inhibitor.

#### Cancer Therapy

Cancer causes abnormal and uncontrolled growth of cells in the human body. The primary tumor is the initial region from where the cancer cells begin to spread. These tumor cells secrete various chemokines to redirect the platelets, immune cells (neutrophils, macrophages, T cells, NK cells) to facilitate their growth and progression in different parts of the body.^314,315^ Circulating tumor cells (CTCs) rapidly spread to the blood and lymph nodes and cause life-threatening metastasis.^316,317^ Therefore, during chemotherapy, delivering the drug at the metastatic site and neutralizing the CTCs in the blood and lymph nodes is crucial. Chimeric antigen receptor T cell immunotherapy (CAR-T),^318,319^ adoptive immunotherapy,^320,321^ immune checkpoint blockade therapy,^322,323^ vaccines, treatment with oncolytic viruses,^324,325^ monoclonal antibodies,^326^ cytokines,^327^ and immunomodulatory treatment^328^ are immunotherapies currently under consideration. However, tumor heterogeneity, immune cell dysfunction, acquired resistance to immunotherapy, and immunotoxicity complicate their clinical translation.^329−331^ Therefore, there is a pressing need to discover and deliver tumor neoantigens to activate the patient’s immune system efficiently.

Incorporating a cancer cell membrane within a CMC mimic provides the required neoantigens, particularly in the case of highly mutagenic tumors. Current treatment options include modifying nano/microparticle surfaces, delivering immunomodulators, and chemo drugs. To resolve the complexity of modification, several CMC mimics with natural biocompatible characteristics have been designed with various combinations of the cell membrane and templates to effectively target primary, metastatic, and CTCs. If required, the cell membrane can be modified further with desired active targeting moieties using lipid insertion^71,234,332,333^ or membrane fusion^97,100,196,198^ to increase its target efficacy toward tumor cells in various organs (brain, breasts, lungs, cervical, colon, pancreas, *etc*.). This section discusses CMC mimics used for cancer treatment, as summarized in Table 3.

RBC membrane-coated PLGA nanoparticles were designed in Zhang’s lab to enhance the low circulation half-life of nanoparticle drug delivery systems by utilizing CD47 receptors on the RBC membrane.^62^ Compared to the PEGylated systems, the half-life of these mimics improved by at least 2-fold, and they remained in circulation for up to 72 h post-injection. Following this work, RBC cell membrane coating of gold nanocages, Fe_3_O_4_ nanoparticles, and PFCs improved their circulation times. Additionally, this enhanced their suitability for bioimaging and phototherapy applications.^265,269,59,68^ Due to the lack of tumors targeting proteins on the RBC membrane, many researchers have attempted modifying with peptides or fusing it with another cell membrane to enhance its targeting efficacy toward specific primary or metastatic tumors. For example, arginyl glycyl aspartic acid (RGD)-modified RBC membrane-coated paclitaxel loaded polycaprolactone (PCL) nanoparticles inhibited the growth of the primary tumor in breast cancer and lung metastasis significantly.^309^ Similarly, brain-targeted peptide (^D^CDX,^234^ T7,^334,335^ and NGR^335−337^)-modified RBC membrane facilitated CMC mimic’s crossing of the blood–brain barrier and improved their ability to target glioma.

Platelet cell membranes gained interest in designing CMC mimics for targeting CTCs due to the effective interaction between P-selectin and CD44 receptors on platelets and tumor cells.^338^ According to these reports, platelet membrane-coated mimics captured and killed CTCs in blood and lymph nodes and inhibited breast cancer metastasis effectively.^87,119^ Further, TNF-related apoptosis-inducing ligand (TRAIL) modified platelet membrane-coated templates also eliminated CTCs effectively. TRAIL additionally activates apoptosis in tumor cells by binding to the death receptors (DR4, DR5) on the cell surface.^213,339,340^ Additionally, coating a hybrid membrane of platelets and leukocytes on commercially available immunomagnetic beads was very effective in isolating pure CTCs from clinical blood samples collected from breast cancer patients, demonstrating the possibility of extending these CTCs for *in vitro* applications and their potential for use in personalized medicine.^99^

**Figure 10:**
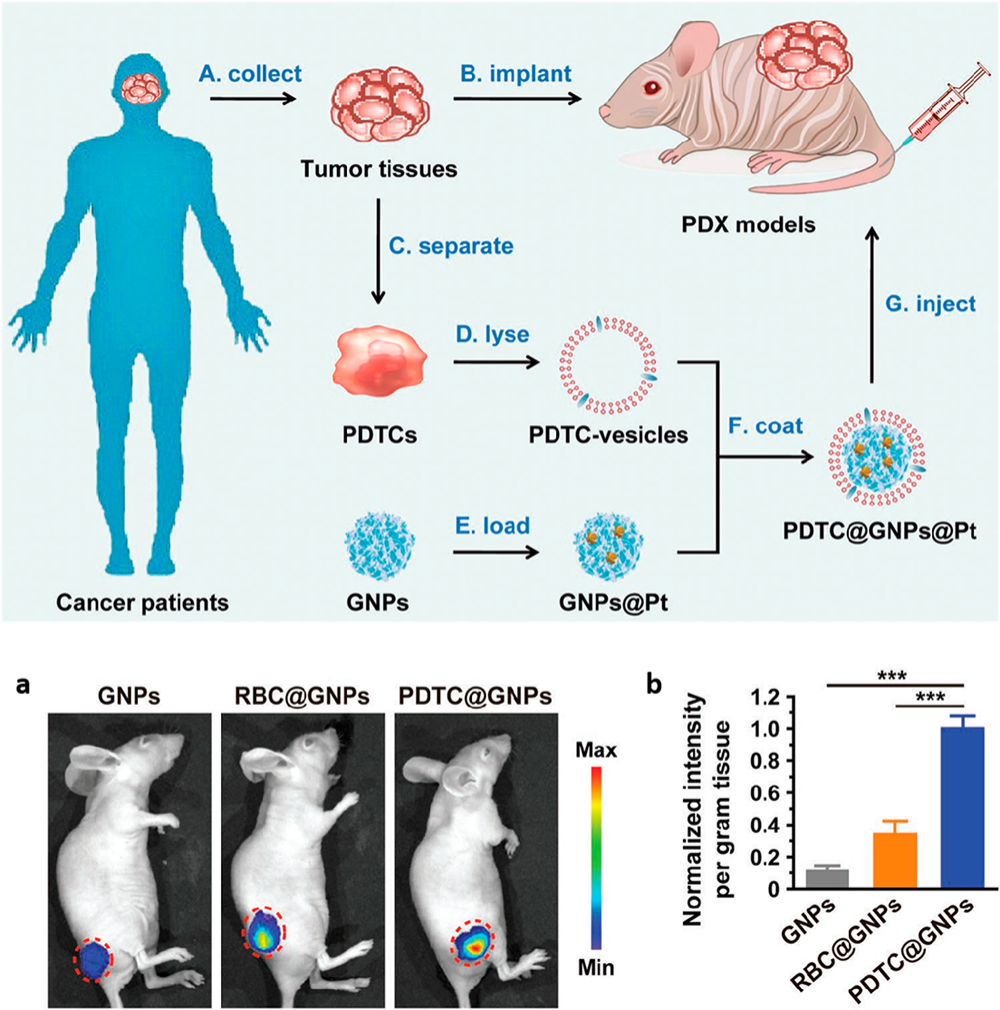
Design of CMC mimics using patient derived tumor cell membrane-coated nanocarriers for personalized cancer therapy. (a) *In vivo* fluorescence images of nude mice bearing PDX at 24 h after intravenous injection of gelatin nanoparticles (GNPs), RBC-coated gelatin nanoparticles (RBC@GNPs), patient-derived tumor cells-coated gelatin nanoparticles (PDTC@GNPs). (b) Normalized *ex vivo* intensity per gram of the PDX tissues in the mice injected with GNPs, RBC@GNPs, and PDTC@GNPs. Reprinted with permission from ref (105). Copyright 2019 John Wiley and Sons.

The cancer cell membrane is known for its homologous targeting abilities attributed to the presence of different adhesion molecules on their surface. These adhesion molecules play an important role in the development of invasive and distant metastasis.^341^ Designing mimics using cancer cell membranes could be a potential strategy to develop personalized tumor-specific therapies or vaccines. In this context, CMC mimics of cisplatin-loaded gelatin nanoparticles coated with the patient-derived tumor cell membrane (head and neck squamous cell carcinoma) (Figure 10) were fabricated and tested for efficacy in a patient-derived xenograft model.^105^ These autologous cell membrane-coated mimics were able to ablate the tumor completely and inhibit tumor recurrence. However, the mismatch of membrane donors and hosts resulted in weaker targeting. Numerous cancer cell membrane-coated mimics using different templates are developed for such homotypic targeting (Table 3).^235,173,219,342,223,174,103,342,175,220,221^

Recently, CMC mimics were redesigned as nanovaccines for cancer. These vaccines combine the cancer cell membrane with an adjuvant that facilitates delivery of tumor-associated antigens to dendritic cells and stimulates the tumor antigen specific-T cells. For example, B16-F10 (murine melanoma cell line) membrane-coated PLGA mimics were designed as nanovaccines for cancer. They incorporated an adjuvant, monophosphoryl lipid A, MPL (FDA-approved LPS derivative) that binds specifically to toll-like receptor 4 to boost the immune response. Additionally, CMC mimics of cancer cell (B16-OVA) membrane and PLGA nanoparticles were formulated as nanovaccines to specifically targeted antigen-presenting cells (APCs) and enhance their uptake to trigger cell maturation. Imiquimod R837, an adjuvant and agonist for toll-like receptor 7 (TLR-7), was preloaded into PLGA particles and coated with a mannose-modified cell membrane.^343^ These mimics effectively inhibited the growth of melanoma tumors when combined with antiprogrammed cell death protein 1 (PD-1) checkpoint blockade therapy. Viruses also have natural adjuvant properties that initiate an immune response. For example, the oncolytic virus replicates inside tumor cells by releasing tumor antigens and causes tumor lysis without affecting healthy cells.^344^ Antigen-presenting cells (APCs) engulf these antigens and redirect dendritic and T cells toward the infected site. Therefore, coating adenovirus serotype-5 virus particles with the melanoma (B16.F10) and lung cancer (LL/2) cell membranes can help explore their properties and treat aggressive melanoma and lung tumor.^214^ Despite coating the virus with different cancer cell membranes, binding efficacy was maximized using homologous and tumor-matched cell membranes.

Macrophages are the most abundant cells in the tumor microenvironment of solid tumors. Interactions between α4 and β1 integrins present on the macrophage surface with the vascular cell adhesion molecule-1 (VCAM-1) present on cancer cells are responsible for progression and metastasis of tumor.^345−348^ Using these interactions, CMC mimics of macrophages (RAW 264.7) and emtansine-loaded liposomes targeted and inhibited lung metastasis in breast cancer models.^129^ In another report, using CCL2/CCR2 chemokines interactions, macrophages were recruited. Quercetin-loaded bismuth selenide nanosystems acted as the templates for mimic assembly.^88^ Post-targeting, nanosystems inhibited primary cancer and lung metastasis by photothermal therapy. Quercetin released inhibited thermoresistant tumors by damaging their heat shock protein 70 (HSP70). Subsequently, several groups have reported the fabrication of CMC mimics with macrophage membranes on various templates that target breast cancer for photothermal therapy.^131,132,139^

Inflammatory neutrophils are activated and directed by the granulocyte-colony stimulating factor (G-CSF) and C–X–C chemokines (CXCL1, CXCL2, CXCL5) toward early premetastatic niche formation.^349,350^ Inspired by this mechanism, neutrophil membrane-coated PLGA nanoparticles loaded with carfilzomib were designed.^55^ These mimics neutralized CTCs, prevented early lung metastasis, and inhibited the progression of already-formed lung metastasis in breast cancer.

Among other immune cells, NK cells can detect and target tumor cells without preactivation. These cells also regulate immune response and T cell activation to kill tumor cells.^144^ The NK cell membrane from murine NK cells and NK-92 cell line-coated on poly(ethylene glycol) methyl ether-*block*-poly(lactide-*co*-glycolide) (mPEG-PLGA) nanoparticles loaded with 4,4′,4″,4‴-(porphine-5,10,15,20-tetrayl) tetrakis (benzoic acid) (TCPP)^150^ were able to polarize M1 macrophages, kill primary tumors, and inhibit the growth of distant tumors. Further, the fusion of the NK cell membrane from NK-92 with liposomes to create NKsomes loaded with doxorubicin demonstrated an excellent tumor homing potential of NKsomes against breast cancer cells.^102^

MSCs have inherent tumor-targeting properties and exhibit immunomodulatory activities.^351,352^ However, biosafety concerns, stability, and reproducibility issues limit their use in clinical applications.^353^ Taking advantage of MSCs mechanism, adipose-derived MSCs membrane-coated Fe_3_O_4_ nanoparticles were constructed as a proof-of-concept to inhibit prostate tumor cells *via* hyperthermia mechanism. Furthermore, bone marrow-derived MSC membranes were used to coat doxorubicin-loaded gelatin nanogels^186^ and UCPNs^185^ to enhance the target efficacy toward cervical tumor cells. The presence of tumor recognition receptors and adhesion molecules on T-cells surfaces has prompted their use for fabricating CMC mimics.^354^

Taking advantage of T-cell receptors, human cytotoxic T-lymphocyte cell membrane-coated paclitaxel-loaded PLGA nanoparticles were designed in combination with low-dose irradiation (LDI) to target gastric tumor cells.^98^ These mimics inhibited gastric tumor growth significantly when used in combination with LDI than mimics alone. Further, an azide-modified T-cell membrane was modified with azide and assembled with a PLGA template for biorthogonal targeting of bicyclo[6.1.0]nonyne (BCN)-modified tumor cells.^158^ Modified cell membrane-coated mimics showed a 1.5-fold higher accumulation around Raji tumor cells.

Dendritic cells (DCs) are the initiators of the primary immune response and capable of activating naïve T cells.^355^ DC-based cancer vaccines have drawn attention in immunotherapy for treating prostate cancer with one of their variants approved by the US FDA.^356^ There is also evidence that immunotherapy could benefit patients with ovarian cancer.^357−359^ Recently, in a clinical phase 1 study for ovarian cancer patients, DC vaccines initiated T-cell responses in only half of the total patients. Their clinical efficacy was affected by low immunogenicity of tumor-associated antigens (TAAs), immunosuppressive tumor-associated microenvironment, restricted migration due to physiological barriers, and downregulation of major histocompatibility complex (MHC).^360−362^ To overcome these limitations and utilize interesting functions of DCs, cell membranes from mature DCs (primed by ovarian cancer cell lysate) were coated on interleukin-2 (IL-2)-loaded PLGA nanoparticles to fabricate mini DCs.^106^ These mini DCs enhanced the activation of the T-cell immune response and effectively inhibited the progression and metastasis of ovarian cancer.

There are reports of CMC mimics designed from the cell membrane of AF to target fibroblast-associated cancer cells. This approach enabled crossing the protective physical barriers built around tumor cells by cancer-associated fibroblasts for delivering anticancer drugs.^363^ Chemically modified nanoparticles are known for their ability to target and kill cancer-associated fibroblasts, prevent biological interactions between tumor and stroma, and enhance chemotherapy.^364^ Based on these reports, CMC mimics were designed using activated fibroblast cell membranes and semiconducting polymeric nanoparticles and compared their efficacy to that of 4T1 cancer cell membrane-coated mimics in breast cancer models.^104^ The AF and 4T1-coated mimics showed superior targeting efficacy for cancer-associated fibroblast and 4T1 cells, respectively, due to their homologous targeting capabilities.

OMVs have the potential to induce the production of antitumor cytokines and trigger the antitumor immune response.^365−367^ Utilizing their mechanism of action, cell membranes of OMVs, and cancer cells were fused to induce both an immune response and increase the homotypic ability. The hybrid cell membrane of *E. coli* DH5α membrane vesicles and B16-F10 cell membrane coating on hollow polydopamine nanoparticles significantly inhibited melanoma growth and stimulated DC maturation in lymph nodes.^200^

#### Inflammation and Immune Diseases

Inflammation is a physiological process that protects the body from harmful stimuli using immune cells, blood vessels, and molecular mediators and promotes tissue repair.^368^ Chronic or uncontrolled inflammation causes diseases like atherosclerosis, ischemic diseases (myocardial infarction, ischemic stroke, hindlimb ischemia), rheumatoid arthritis, and acute liver failure. Modulation of inflammatory responses to balance the immune homeostasis helps to overcome the disease progression.^369^ Some of the inflammation-related cells that play a vital role in shaping its microenvironment are neutrophils, NK cells, macrophages, lymphocytes, platelets, and stem cells. These cells are in their resting state during circulation but are activated by cytokines or chemokines during inflammation and migrate to the infected site.^370,371^ Therefore, designing CMC mimics using these cells has considerable potential to treat inflammatory diseases.

Atherosclerosis is a condition caused by the accumulation of lipids, cholesterol, and fibrous elements in the artery wall that restricts blood flow.^372^ The primary challenge of this disease is it is asymptomatic until the very late stages. Surgically stenting the artery is often the preferred intervention route. However, this can lead to potential side effects such as restenosis and stent thrombosis, eventually triggering neointimal hyperplasia.^373,374^ Using a noninvasive strategy to image and monitor plaque development would be the preferred mode of treatment, as platelets are responsible for hemostasis in the body and are involved in atherogenesis.^375−378^ CMC mimics assembled with their cell membranes may provide a viable alternative to the existing line of treatment. The platelet membrane-coated PLGA nanoparticles loaded with docetaxel were reported for restenosis therapy.^93^ Their results concluded that CMC mimics localized better than the drug alone at the plaque site and inhibited neointima growth. Further, the MRI-based platelet membrane-coated PLGA nanoparticles localize better at the plaque-forming and atherosclerosis areas than the PLGA or RBC membrane-coated PLGA nanoparticles, providing crucial information for managing atherosclerosis.^123^ The nonthrombogenic and stent-free restenotic therapy was also treated using platelet membrane-coated nanoclusters of poly(amidoamine) and polyvalerolactone (PAMAM-PVL).^122^ These dendritic, unimolecular nanoclusters were preloaded with an endothelium-protective epigenetic inhibitor (JQ1) before coating. Comparing the efficacy of the JQ1 and rapamycin (endothelium-toxic status quo drug) showed up to a 60% reduction of neointimal hyperplasia. However, rapamycin impairs the endothelial recoverage, while JQ1 protects the endothelial coverage in the inner artery wall. Therefore, these noninvasive platelet membrane-coated mimics loaded with MRI contrast agents or endothelium-protective inhibitors or drugs have the potential for live imaging to assess, prevent, and manage the development of atherosclerosis at an early stage.

Restricted blood flow in blood vessels resulting in tissue damage or dysfunction is a characteristic of ischemic disease.^379^ Mesenchymal stem cells are a promising candidate for their treatment as they can interact effectively with the stromal-derived factor (SDF) overexpressed in ischemic tissue through their CXCR4.^380,381^ Researchers have tried to overexpress the CXCR4 receptor on stem cells to increase its efficiency. For example, they bioengineered human adipose-derived stem cell (hASCs) membranes to overexpress CXCR4 receptors for coating PLGA nanoparticles loaded with vascular endothelial growth factor (VEGF). These particles showed a higher accumulation at the ischemic site than the hASC-coated mimics.^227^ Likewise, a neural stem cell membrane overexpressing CXCR4 was coated on PLGA nanoparticles loaded with glyburide for testing in ischemia stroke models.^188^ Even in the injured brain, these CMC mimics could effectively cross the blood–brain barrier for drug delivery. PLGA microparticles loaded with secretomes were coated with cardiac stem cell membrane for myocardial infarction.^187^ Their functional efficacy was comparable to cardiac stem cell therapy and allowed for surgical transfer intramyocardially.

Acute liver failure causes deterioration of liver function and requires liver transplantation to cure the patient.^382^ Stem cell therapy can be a promising treatment for liver failure, as MSCs secret anti-inflammatory factors reducing inflammation and promote healing.^383^ Cultured MSCs with 20 μm in diameter are larger than the width of the microcapillaries of the lung;^384^ therefore, intravenously infused MSCs are short-lived and easily filtered by the lungs and do not reach the liver.^385^ Thus, using RBC membrane-coated PLGA nanoparticles loaded with MSCs regenerative factors of 200 nm in size resolved this size issue.^86^ The small size of the mimics helped them pass through the lungs and reach the liver, and additionally, the RBC membrane coating prolonged their circulation time.

Rheumatoid arthritis (RA) is another autoimmune disorder that leads to joint damage and disability. Current treatment focuses on targeting the inflammatory responses such as inhibiting the interleukin (IL)-1 and tumor necrosis factor-alpha (TNF-α).^386,387^ Various chemoattractant are known to promote neutrophil migration into the joints during RA.^388^ Microvesicles produced by neutrophils can readily enter cartilage and protect joints.^389^ Therefore, neutrophil membrane-coated PLGA nanoparticles were designed.^66^ These mimics neutralized pro-inflammatory cytokines (IL-1β and TNF-α), suppressed synovial inflammation, targeted cartilage matrix, and protected chondrocytes against damage. Apart from neutrophils, other cell membrane-like T cells, dendritic cells, macrophages, and monocytes are present in the primary stage of RA, working together for its progression. Therefore, CMC mimics using the membrane of these cells have the potential to show stealth properties for the treatment of RA.^390,391^

#### Infectious Diseases

Pathogens, viruses, and bacteria cause infectious diseases by interacting and penetrating the host cell membrane.^392,393^ A lack of effective drugs, specific treatment options, and increasing drug-resistant strains caused by overuse of antibiotics are the main challenges to their treatment. CMC mimics can target viruses, bacteria, and multidrug-resistance bacteria and absorb toxins.

##### Viral Infection

**Figure 11:**
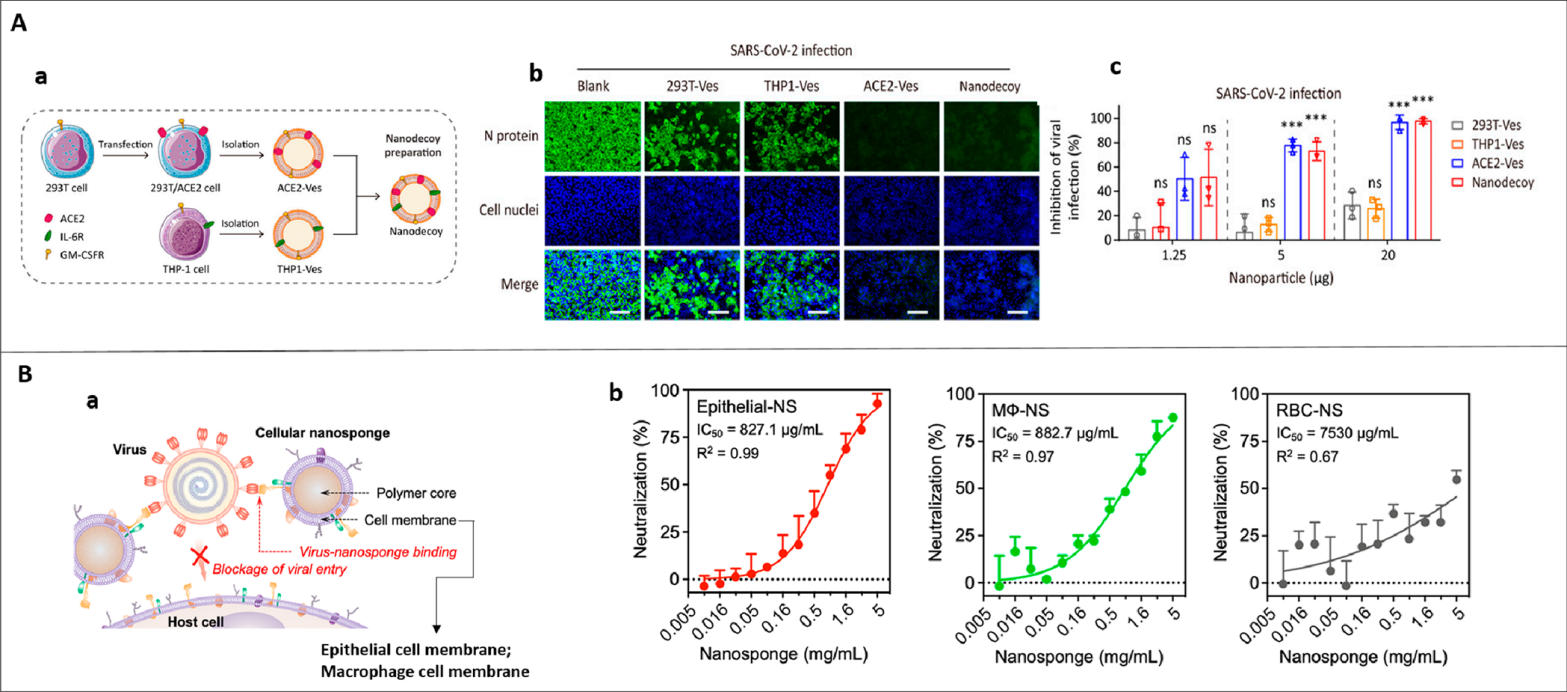
Potential of CMC mimics in treating COVID-19 infection. (A) (a) Preparation of nanodecoys using fused cell membrane vesicles derived from genetically edited 293T/ACE2 and THP-1 cells. (b) Immunofluorescence images of SARS-CoV-2-infected Vero-E6 cells after treatment with nanodecoys, scale bar = 100 μm, cell nuclei: DAPI (blue), N protein of SARS-CoV-2: Alexa 488 (green). (c) Inhibitory activity of nanodecoys against SARS-CoV-2 on Vero-E6 cells. Reprinted with permission under a Creative Commons CC-BY License from ref (397). Copyright 2020 United States National Academy of Sciences. (B) (a) Preparation of nanosponges using epithelial cell membrane and macrophage cell membrane-coated PLGA nanoparticles. (b) Neutralization of SARS-CoV-2 infection by epithelial nanosponges, macrophage (MΦ) nanosponges, RBC nanosponges (control), using SARS-CoV-2 on Vero-E6 cells. Reprinted with permission from ref (400). Copyright 2020 American Chemical Society.

Infections caused by viruses are the most difficult to treat, as viruses do not follow a regular cell division process for their growth. They replicate by binding to the receptors on the host cell membrane and infusing genetic material inside them. CMC mimics can be potential alternatives to neutralizing virus-caused infections. Many cells are rich in virus binding receptors, and fabricating CMC mimics from the cell membrane of these cells diverts viruses from host cells. This mechanism is employed for treating infections caused by the influenza virus, zika virus, and human immunodeficiency virus (HIV), and recently by COVID-19 (Figure 11).

The surface of the influenza virus is rich in hemagglutinin, a glycoprotein that has a high affinity toward sialic acid residues present in cells.^394^ RBCs membrane is rich in sialic acid and glycoproteins. Designing CMC mimics using their cell membrane can favorably interact with the influenza virus shown by RBC membrane-coated PLGA nanoparticles.^74^ These mimics bind efficiently with the influenza virus and form clusters that can be readily isolated *in vitro* by magnetic extraction.

COVID-19 is a viral infection caused by severe acute respiratory syndrome coronavirus 2 (SARS-CoV-2). The spike protein (S) of SARS-CoV-2 consists of S1 and S2 subunits. The S1 subunit engages human angiotensin-converting enzyme II (ACE2) for binding with the host cells, and the S2 subunit facilitates the entry and fusion of the virus within the host cells. Preclinical and clinical studies report that monoclonal antibodies targeting interleukin 6 (IL-6) and granulocyte-macrophage colony-stimulating factor (GM-CSF) can prevent the infection caused by SARS-CoV-2.^395,396^ Based on these reports, the human embryonic kidney 293T cell membrane genetically engineered to express ACE2 receptor and monocyte THP-1 cell membrane with abundant cytokine binding receptors were combined to form vesicles.^397^ These fused vesicles effectively bind and neutralize IL-6 and GM-CSF, suppressing the immune disorders and lung injury in the acute pneumonia mouse model. The ACE2 receptor on SARS-CoV-2 also binds to CD147 expressed on the host cells, human alveolar epithelial type II cells, and human macrophages.^398,399^ PLGA nanoparticles coated with their cell membrane effectively target SARS-CoV-2.^400^ Overall epithelial and macrophage-based CMC mimics are preferable for inhibition and neutralization of SARS-CoV-2 (Figure 11).

Zika virus is a mosquito-borne flavivirus, transmitted by Aedes mosquitos (*Aedes albopictus*, *Aedes aegypti*).^401,402^ They can easily pass physiological barriers like the brain–blood barrier and placental–blood barrier, causing fetal microcephaly and other neurological complications.^403,404^ In general, the nanoparticles cannot enter the immune-privileged sites.^405^ Coating gelatin-nanoparticles with the *Aedes albopictus* (C6/36) cell membrane circumvents this limitation.^228^ These CMC mimics divert the Zika virus away from the fetal brain, suppress fetal microcephaly in pregnant mice, negate virus-induced degenerative changes, prevent replication, and improve overall survival rate.

HIV infects leukocytes *via* interaction between glycoproteins on its surface (gp120) and cluster of differentiation 4 (CD4) receptor, C–C chemokine receptor type 5 (CCR5), or CXCR4 coreceptors on CD4^+^ T cells.^406^ CD4^+^ T-cell membrane-coated PLGA nanoparticles effectively treated two distinct HIV strains: X4 and R5.^159^ These mimics neutralized the strains and prevented the HIV-1 from binding to and entering the healthy CD4^+^ T cells.

##### Bacterial Infection

Bacteria show both positive and negative impacts on the human body. While probiotic bacteria aid the digestive process, other bacterial strains (Gram-positive: *S. aureus* and Gram-negative: *E. coli, K. pneumonia*) cause mild to severe infections and host cell death. Additionally, excessive use of antibiotics results in drug-resistant bacterial strains (*e*.*g*., methicillin-resistant *S. aureus* (MRSA), carbapenem-resistant *K. pneumonia* (CRKP)) in the human body, posing additional therapeutic challenges. This section highlights antimicrobial activity and toxin neutralization using bacterial- and cell membrane-coated mimics.

The α-toxins are a class of pore-forming toxins secreted by bacteria (*E. coli, S. aureus*, MRSA, *etc.*) that create pores in the host cell membrane, causing cell lysis.^407^ RBCs and platelets express several surface markers (*e.g*., glycophorin A in RBCs,^112^ toll-like receptors in platelets^119^) that readily interact with such pathogens. Using these receptors, RBC membrane-coated PLGA nanoparticles sequestered α-toxins^115^ and platelet membrane-coated PLGA nanoparticles^93^ delivered vancomycin to MRSA252. Platelet membrane-coated particles loaded with vancomycin (PNP-Vanc) showed superior efficacy, specificity, and retention due to the presence of platelet-specific serine-rich (SraP) adhesion sites on MRSA252.^395^ Similarly, RBC membrane-coated vancomycin-loaded redox responsive hydrogels absorbed α-toxins and killed MRSA.^114^ The intracellular reducing environment of the bacteria triggered vancomycin release from the hydrogels. Other examples include acoustic nanorobots of gold nanorods coated with the hybrid membrane (platelet and RBC). These fuel-free nanorobots accelerated toxin neutralization and removal of bacteria (MRSA USA300).^196^ RBC membrane coating on carbon nanotubes-based field transistors resulted in rapid detection of several pore-forming toxins. These biomimetic nanosensors could quantitatively detect live pathogens without involving traditional colony-counting methods.^75^

Homotypic targeting utilizes the membrane of the targeted pathogen for specificity. For example, bovine serum albumin nanoparticles coated with CRKP bacterial membranes enhanced the immune response by secreting cytokines from macrophages and activating dendritic cells.^193^ Similarly, *S. aureus* membrane-coated PLGA nanoparticles showed superior targeting efficacy toward *S. aureus*-infected macrophages.^192^ These mimics actively targeted all organs except the liver and showed improved efficacy in kidneys and lungs prone to a higher risk of *S. aureus* infections. Similarly, *E. coli* membrane-coated gold nanoparticles coated with *E. coli* membrane removed *E. coli* bacterial infection.^94^ They also activated dendritic cells in lymph nodes and increased the production of IFN-γ and IL-17, but not IL-4 generating type 1 T-helper cell (Th1) and type 17 T-helper cell (Th17)-based cell responses against bacterial infections.

## Current Challenges

Ingraining complex biological functionalities in delivery systems is a significant outcome of coating with cell membranes that differentiates them from synthetic mimics. Throughout this review, we have emphasized why these CMC mimics utilizing surface functional properties of cells are better suited for therapeutic applications over their synthetic counterparts. Thus far, fabrication and *in vitro* and *in vivo* evaluation studies on CMC mimics are limited to the lab setting. However, the actual therapeutic dose of materials required and conditions for clinical studies are higher and stringent. In this context, it is vital to have standardized protocols and well-established characterization tools for scale-up and GMP production of these mimics to maintain quality and reduce batch-to-batch variability. Herein, we discuss some of the challenges associated with their clinical translation.

(1) Large-scale expansion: Cell membrane isolation requires at least a 100 million cells that maintain their phenotype, purity, and quality while passaging. A standardized and well-established cell culture protocol specific to each cell type is essential for large-scale production. In this regard, one can benefit from the existing well-established biomanufacturing platforms using 3D bioreactors (like stirred-tanked bioreactor, WAVE bioreactor, *etc.*) for the ultralarge scale-up of stem cells, T cells, and dendritic cells.^411−414^
(2) Cell membrane yield: Lab-scale procedures for cell membrane isolation are multistep and specific to each cell type and may result in loss of sample, functional receptors, and nuclear/mitochondrial/cytosol contamination. Therefore, there is a requirement for an established protocol with minimal manual steps for cell membrane isolation with high yield and purity for various cells, especially for nucleus-containing cells.
(3) Assembly of CMC mimics: In CMC assembly, it is vital to control the cell membrane layers coated onto each template while achieving a homogeneous coating. The physiological effects of differences in membrane layers on CMC mimics remain unexplored. Using automated technology to improve the coating efficiency and avoid uneven coating can be a viable alternative. For example, assembly of RBC-coated CMC mimics using the microfluidic electroporation technique resulted in uniform-sized mimics.^57^
(4) Long-term storage: Optimizing long-term storage conditions and membrane stability of CMC mimics are critical to improve their shelf life. Post-lyophilization isolated cell membranes can be stored in cold conditions and resuspended in buffers before use. However, shelf life studies to determine the stability and functional efficacy of isolated cell membranes remain unexplored.
(5) Quantitative evaluation of CMC mimics: An in-depth quantitative characterization of CMC mimics is essential to avoid batch-to-batch variability in their biological efficacy. These include the number of templates coated evenly with and without the cell membrane and the amount of transmembrane protein translocated on the mimics in the correct orientation.
(6) Quality control: A standard quality control criteria must be defined to ensure that the cell membranes are free of contamination like viruses, bacteria, or pyrogens. Additionally, removal of denatured proteins from the CMC mimics avoids potential immune responses to endogenous antigens. Also, every assembly step (isolation of cell membrane, synthesis of template, fabrication of CMC mimics) should be carried out under sterile conditions to avoid chemical and biological contamination and maintain GMP requirements.
(7) Unwanted proteins on the CMC mimics: There are several proteins present on the cell membrane. Some are accountable for effective targeting and evading immune responses, and others are involved in interacting with the host environment affecting biodistribution, immune response, and toxicity profile. Optimizing protocols to selectively retain proteins of interest and remove unwanted proteins from the cell membrane can enhance the CMC performance and remains to explore.
(8) Surface modification of cell membrane: Numerous membrane modification strategies are known, but not all of them offer proper orientation, linkage strength, and conserve membrane protein functionality. For example, noncovalent modifications protect the membrane protein functionality, but the interactions are weaker in linkage strength.^415^ Conversely, covalent bonding with a template is robust, but there is a risk of altering the natural membrane functionality and compromising the protein profiles. Changes in the ζ potential only primary measure the membrane modification,^333^ creating a necessitous gap in qualitative and quantitative evaluation techniques. It is also complicated to observe small-molecule conjugation on the cell membrane and evaluate the overall impairment.
(9) Autologous cells: For designing CMC mimics, most studies have utilized immortal cell lines. However, certain cell types (like leukocytes) can be heterogeneous and induce hemolysis during a blood transfusion. In such cases, autologous cells are the most suitable option, but would require screening of the donors to prevent the use of allogeneic cells as membrane sources.

## Vocabulary

**Biointerface**: point of interaction between nano/microparticles and the surrounding cells
**personalized medicine**: modifying disease treatment to suit the patient’s genetic profile
**autologous therapy**: utilizing patient-derived cells for disease treatment
**tumor microenvironment**: cellular environment surrounding tumors consisting of immune cells, blood vessels, and extracellular matrix that facilitates their growth
**receptor**: proteins present on the cell surface that binds selectively to a ligand to transmit signals.
